# Phosphorescent Iridium Hydrazinonicotinic Acid (HYNIC) Complexes That Bind to Prostate Specific Membrane Antigen: Potential Photodynamic Therapy of Prostate Cancer

**DOI:** 10.1002/chem.71011

**Published:** 2026-04-17

**Authors:** La'El Kimchi, Emily R. McGowan, Katherine A. Morgan, Jonathan M. White, Trevor A. Smith, Stacey E. Rudd, Paul S. Donnelly

**Affiliations:** ^1^ School of Chemistry and Bio21 Molecular Science and Biotechnology Institute University of Melbourne Melbourne Victoria Australia; ^2^ School of Chemistry University of Melbourne Melbourne Victoria Australia

**Keywords:** bioinorganic chemistry, iridium, molecular targeting, phosphorescent, photodynamic therapy

## Abstract

Phosphorescent metal complexes have the potential to be used for photodynamic detection of tumor margins in surgery and photodynamic therapy (PDT). In this work, iridium(III) complexes are prepared with two cyclometalating ligands, either phenylpyridine (ppy) or phenylisoquinoline (piq), and one 6‐hydrazinonicotinic acid (HYNIC) ancillary ligand to give [Ir(ppy)_2_(HYNIC)]^+^ and [Ir(piq)_2_(HYNIC)]^+^. The extended conjugation in [Ir(piq)_2_(HYNIC)]^+^ results in a significant redshift in the absorption and emission properties. Both [Ir(ppy)_2_(HYNIC)]^+^ and [Ir(piq)_2_(HYNIC)]^+^ generate singlet oxygen upon irradiation with light in the presence of oxygen. Irradiation of [Ir(piq)_2_(HYNIC)]^+^ (*λ*_exc_ = 420 nm) results in the production of hydroxyl and superoxide radicals. The carboxylic acid functional group in HYNIC has been used to attach a lysine‐ureido‐glutamatic acid pharmacophore that selectively binds to prostate specific membrane antigen (PSMA) to give HYNIC–PSMA. PSMA is an enzyme that is overexpressed in prostate cancer. HYNIC–PSMA was used to prepare [Ir(ppy)_2_(HYNIC–PSMA)]^+^ and [Ir(piq)_2_(HYNIC–PSMA)]^+^. Both complexes bind to cells that overexpress the PSMA enzyme. [Ir(piq)_2_(HYNIC‐PSMA)]^+^ is nontoxic to cells in the dark, but irradiation with visible light results in a dose‐dependent cytotoxicity. These complexes have the potential to be of use to identify tumor margins, to guide robot‐assisted surgical resection of tumors, as well as for molecularly targeted PDT.

## Graphical abstract

A phosphorescent iridium(III) complex has been prepared with a 6‐hydrazinonicotinic acid (HYNIC) ligand tethered to a lysine‐ureido‐glutamatic acid pharmacophore, which binds to an enzyme, called prostate‐specific membrane antigen (PSMA), which is overexpressed in prostate cancer. The new complex has the potential to be used for molecularly targeted photodynamic detection and photodynamic therapy (PDT) of prostate cancer.

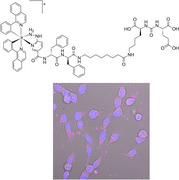

## 1 Introduction

Surgical resection of tumors requires accurate identification of tumor margins, and often the surgeon relies on palpation and visual inspection under ambient light. The use of fluorescent molecules to differentially stain tissue and fluorescence imaging techniques can improve image contrast [1]. Dyes such as indocyanine green and methylene blue have proved useful to improve visual contrast within tissue, but do not selectively bind to the tumor tissue [1, 2]. It is possible to improve specificity in identifying tumour tissue by developing tumor‐targeting fluorescent probes by conjugating dyes to either antibodies or molecules that bind selectively to receptors that are overexpressed in tumors [3, 4, 5].

Tumor resection with robotic‐assisted surgery, where a surgeon controls robotic arms to perform surgery through small incisions, is becoming more common in clinical practice. Robot‐assisted surgery can be augmented using gamma‐emitting technetium‐99^m^ tracers and “drop‐in” gamma detectors to enable single photon emission computed tomography (SPECT) imaging to help identify tumor tissue [6]. In principle, the use of fluorescence imaging offers the potential of better resolution than SPECT imaging, albeit with reduced sensitivity. Most robot‐assisted clinical studies use traditional organic fluorophores such as fluorescein, indocyanine green, sulfo‐Cy5, and IRDye800CW [4, 7, 8].

**FIGURE 1:**
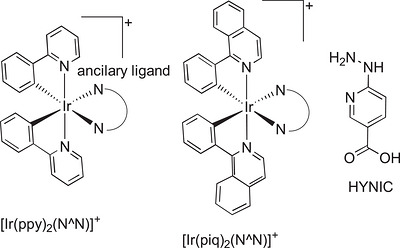
Chemical structures of [Ir(ppy)_2_(N^^^N)]^+^, [Ir(piq)_2_(N^^^N)]^+^, and HYNIC.

This work focuses on the potential to use phosphorescent organometallic iridium(III) complexes as the light emitting species in place of traditional organic dyes. When compared to conventional organic fluorophores, phosphorescent organometallic iridium(III) complexes with two cyclometalating bidentate ligands (C^N) such as 2‐phenylpyridine (ppy) or 1‐phenylisoquinoline (piq) and one bidentate nitrogen donor “ancillary ligand (N^N)” (Figure 1) have large Stokes’ shifts, long‐lived phosphorescence and are largely resistant to photo‐bleaching suggesting potential advantages in designing interoperative fluorescence probes to guide surgical resection of tumours [9]. Strong spin–orbit coupling in iridium(III) complexes, due to the presence of the heavy metal atom, promotes intersystem crossing and population of a triplet metal to ligand charge transfer state (^3^MLCT) that results in emission with quite long phosphorescence emission lifetimes [10, 11]. The ^3^MLCT excited state can interact with oxygen to form cytotoxic singlet oxygen (^1^O_2_), providing the added advantage of potential photodynamic therapy (PDT) applications [11, 12, 13, 14].

PDT involves activation of a molecule by light to initiate a therapeutic effect. PDT is used to treat certain forms of cancer, some skin conditions, and age‐related macular degeneration. It is feasible that robotic surgery equipment could be augmented for the endoscope camera to involve fibre optic‐delivered lasers that could be used for minimally invasive PDT to be coupled with photodynamic detection [6]. One of the advantages of PDT when compared to traditional chemotherapy is that the photosensitizer and light are required to induce cytotoxicity and, in some instances, even invoke an immune response [14]. The mechanism of action of PDT is a complicated process but generally falls into two categories: type I processes involve photoinduced electron transfer to form superoxide (O_2_^•−^) or hydroxyl radical (HO^•^); type II involves energy transfer from the photosensitizer to ground state oxygen (^3^O_2_) to form singlet oxygen (^1^O_2_) [12]. The most commonly used and investigated photosensitizers are derived from porphyrins or phthalocyanines, where overall efficacy is limited by short‐lived excited states and rapid photobleaching. In contrast to purely organic photosensitizers, iridium(III) complexes offer many different excited‐state electronic configurations that can be exploited in oxygen‐independent and oxygen‐dependent pathways [12].

In this work, we demonstrate that 6‐hydrazinonicotinic acid (HYNIC) can be used as a bidentate ancillary ligand to form luminescent iridium(III) complexes (Figure 1). The HYNIC ligand has been used extensively as a bifunctional chelator in the design of molecularly targeted technetium‐99m complexes for applications in SPECT imaging [15]. Coordination of HYNIC to technetium(V) involves multiple bonds between the terminal hydrazine functional group and the metal, as well as the pyridyl nitrogen. The carboxylate functional group of HYNIC can be used to attach tumor‐targeting peptides [16, 17]. The versatility of HYNIC derives from the ability to act as an “unnatural” amino acid for conjugation to a targeting peptide using solid‐phase peptide synthesis [18]. Here, HYNIC is attached to a lysine–ureido–glutamatic acid pharmacophore that binds selectively to prostate specific membrane antigen (PSMA), a zinc metalloenzyme that is overexpressed in metastatic prostate cancer. The protease active site of PSMA contains two zinc(II) ions that reside within a deep hydrophobic tunnel (20 Å). The lysine–ureido–glutamatic acid pharmacophore is separated from the HYNIC chelator by two hydrophobic D‐phenylalanine residues and 8‐aminooctanoic acid (Aoc) to provide a metabolically stable hydrophobic linker. This combination of linkers with the lysine‐ureido‐glutamatic acid pharmacophore is used in a copper‐64/67 imaging and therapeutic agent that is in human clinical trials [19, 20, 21]. The new HYNIC conjugate is used as an ancillary ligand to form phosphorescent iridium(III) complexes.

## 2 Results and Discussion

**SCHEME 1:**
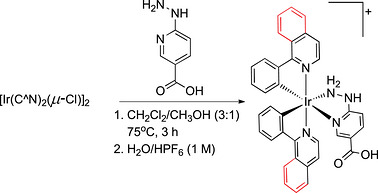
Synthesis of [Ir(C^^^N)_2_(HYNIC)]^+^, where C^^^N is either ppy or piq (red).

Both [Ir(ppy)_2_(HYNIC)]^+^ and [Ir(piq)_2_(HYNIC)]^+^ were prepared by reacting the appropriate iridium dimer precursor, [Ir(C^N)_2_(*μ*‐Cl]_2_, with 2.5 equivalents of HYNIC in a mixture of dichloromethane and methanol (Scheme 1). Acidification of the reaction mixture with HPF_6_ allowed for isolation of the monocationic complexes as their PF_6_ salts. The complexes were characterised by NMR spectroscopy and electrospray ionization mass spectrometry. Analysis of the complexes by NMR spectroscopy demonstrated that the complexes were stable in solution under ambient light for at least 24 h with no signs of ligand substitution.

**FIGURE 2:**
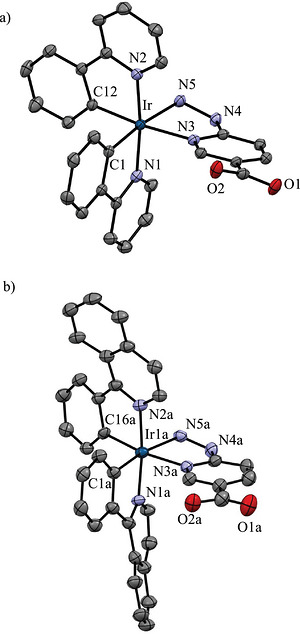
ORTEP representations (50% ellipsoids) of the x‐ray crystal structures of [Ir(ppy)_2_(HYNIC)] (a) and the cation present in [Ir(piq)_2_(HYNIC)]Cl.CH_3_CN.(CH_3_CH_2_)_2_O. (b) Hydrogen atoms, anions, and solvent molecules omitted for clarity.

[Ir(ppy)_2_(HYNIC)], isolated as the charge neutral complex with the coordinated HYNIC ligand deprotonated at the carboxylic acid functional group and [Ir(piq)_2_(HYNIC)]^+^, isolated as the monocationic chloride salt, were also characterized by single crystal x‐ray crystallography (Figure 2).

In both complexes, the iridium(III) is in a distorted octahedral C_2_N_4_ environment with the pyridyl functional groups of the cyclometalating ligand *trans* to each other and the organometallic Ir─C bonds *cis* to each other. Both Δ and Λ enantiomers are present in the unit cell. In [Ir(ppy)_2_(HYNIC)] the Ir─C bonds (2.001(3) and 2.020(3) Å) are shorter than the Ir─N_pyridyl_ bonds (2.044(3) and 2.047(3) Å) of the cyclometalating ppy ligand. The analogous bond lengths in [Ir(piq)_2_(HYNIC)]^+^ are similar. In both complexes, the hydrazine functional group of the HYNIC ligand coordinates as a charge‐neutral donor and forms a 5‐membered chelate ring with the pyridyl group of HYNIC. In both the complexes, the Ir─N_hydrazine_ bonds (Ir‐N5 2.178(3); Ir1a‐N5a 2.187(4) Å) are longer than the Ir─N bond with the pyridyl group of the HYNIC ligand (Ir‐N3 2.134(3); Ir1a‐IrN3a 2.121(4) Å). The N─N bond distances within the hydrazine functional group, N4‐N5 1.442(4) Å in [Ir(ppy)_2_(HYNIC)]^+^ and N4a‐N5a 1.423(6) Å in [Ir(piq)_2_(HYNIC)]^+^, are consistent with single bond character and the hydrazine functional group coordinating as a charge‐neutral donor. The coordination of the hydrazine group as an NH_2_─NH_2_─pyridyl charge‐neutral donor is different from how HYNIC coordinates in rhenium(III) and technetium(III) complexes, where the N─N bond lengths are indicative of multiple bond character within the deprotonated hydrazine group [22]. For example, in a rhenium(III) complex with two HYNIC ligands, [ReCl_3_(*k* ^1^‐HYNIC + H^+^)(*k* ^2^‐HYNIC)], one HYNIC ligand acts as a monodentate diazenido(‐1) ligand, which is protonated at the pyridyl nitrogen, and the other HYNIC ligand acts as a chelating pyridyl‐diazene neutral donor. In both cases, extensive multiple bond character results in significant shortening of the N─N bond distances (1.26(1) and 1.34(1) Å) of the respective hydrazine fragments [22].

**FIGURE 3:**
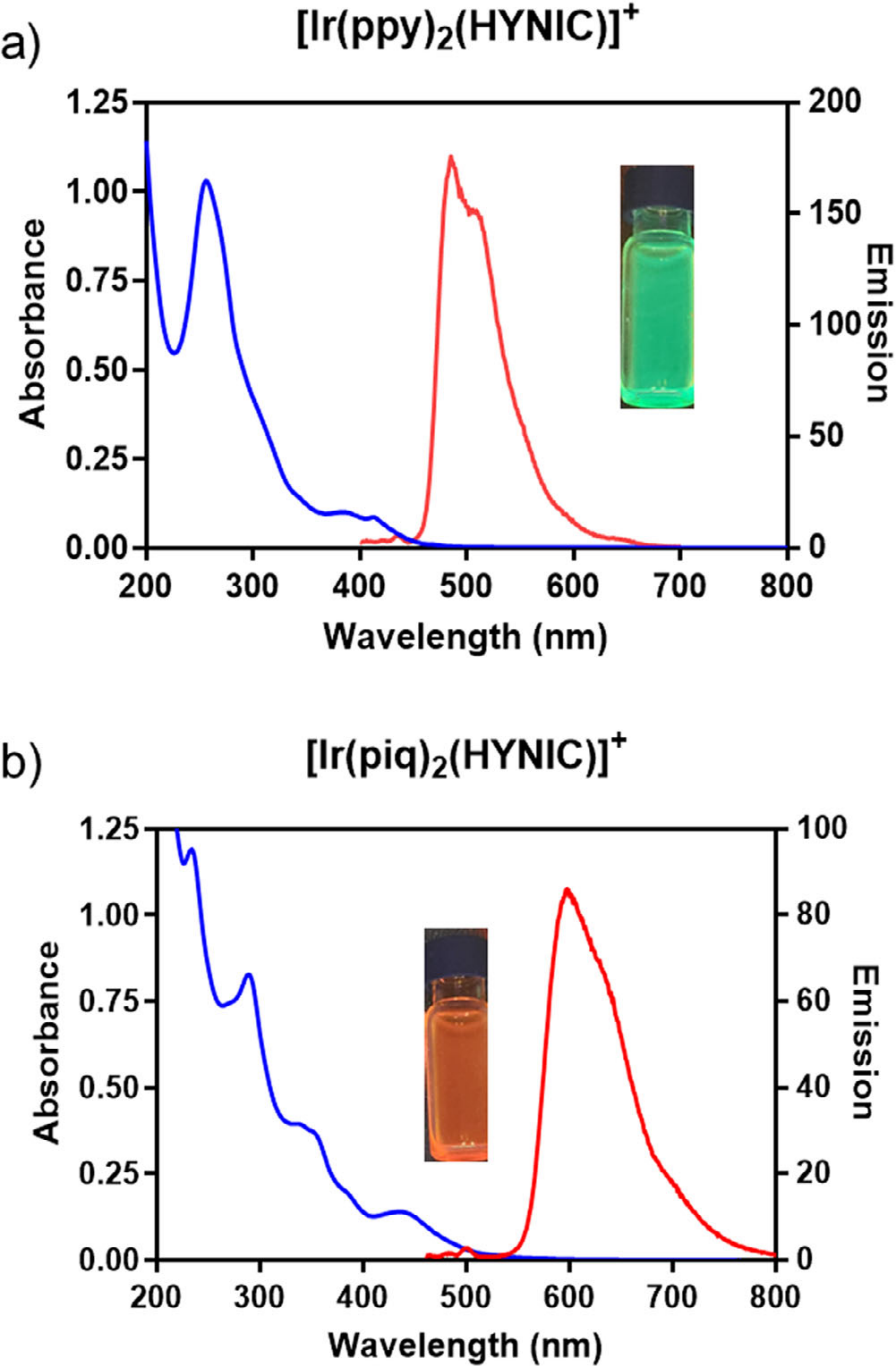
Absorption and emission spectra of (a) Ir(ppy)_2_(HYNIC)]^+^ and (b) [Ir(piq)_2_(HYNIC)]^+^. Inset photographs showing the phosphorescence of solutions of each complex following irradiation, *λ*_exc_ = 380 nm.

The electronic absorption spectrum of [Ir(ppy)_2_(HYNIC)]^+^ has a *λ*_max_ at 255 nm, and the spectrum of [Ir(piq)_2_(HYNIC)]^+^ contains maxima at *λ*_max_ = 234 and 289 nm that can be attributed to π–π* ligand‐centred (^1^LC) transitions within the cyclometalating ppy and piq ligands, respectively (Figure 3). An absorption maximum at lower energy, *λ*_max_ = 383 nm in [Ir(ppy)_2_(HYNIC)]^+^, is attributed to spin‐allowed metal‐ligand charge transfer transitions (MLCT) [23, 24]. The presence of the extended conjugation in the piq ligand in [Ir(piq)_2_(HYNIC)]^+^ results in a bathochromic shift in the MLCT to *λ*_max_ = 435 nm. Apparent vibronic contributions in the absorbance spectrum of [Ir(piq)_2_(HYNIC)]^+^ are consistent with increased charge mixing between the cyclometalating piq π* orbital and ancillary ligand‐based HYNIC π* orbital [24, 25].

Both [Ir(ppy)_2_(HYNIC]^+^ and [Ir(piq)_2_(HYNIC)]^+^ are emissive at room temperature when dissolved in acetonitrile (Figure 3). The emission from [Ir(ppy)_2_(HYNIC)]^+^ is centered at *λ*_em_ = 485 nm (*λ*_exc_ = 380 nm) with a vibronic shoulder at λ_em_ = 511 nm due to charge mixing between the ^3^MLCT and ^3^LC states [24]. The bright red emission from [Ir(ppy)_2_(HYNIC)]^+^ is centred at *λ*_em_ = 597 nm (*λ*_exc_ = 440 nm) and is broad and consistent with emission from a ^3^MLCT [26]. The emission quantum yield (*Φ*) and excited state lifetime of [Ir(ppy)_2_(HYNIC)]^+^ in deoxygenated acetonitrile are *Φ* = 0.26, *τ* = 2.58  µs and for [Ir(piq)_2_(HYNIC)]^+^ *Φ* = 0.20, *τ* = 2.26 µs. The complexes are emissive in phosphate‐buffered saline (PBS) (99:1 PBS/DMSO (v/v), pH = 7.4) but with lower quantum yields, (*Φ* = 0.31 for [Ir(ppy)_2_(HYNIC)]^+^, and *Φ* = 0.009 for [Ir(piq)_2_(HYNIC)]^+^).

The ability of [Ir(ppy)_2_(HYNIC)]^+^ and [Ir(piq)_2_(HYNIC)]^+^ to generate singlet oxygen following activation by light (*λ* = 365 nm) was measured in aerated acetonitrile using 1,3‐diphenylisobenzofuran (DPBF) as a chemical trap for ^1^O_2_ [27]. Solutions of the complexes in the presence of DPBF were subject to irradiation with light (*λ*_exc_ = 365 nm). The decrease in absorption at *λ*_abs_ = 411 nm (due to loss of DPBF) was measured and plotted against cumulative irradiation time. The singlet oxygen quantum yields (*Φ*_∆_) are *Φ*_∆_ = 0.4 and *Φ*_∆_ = 0.8 for [Ir(ppy)_2_(HYNIC)]^+^ and [Ir(piq)_2_(HYNIC)]^+^, respectively.

The ability of [Ir(ppy)_2_(HYNIC)]^+^ and [Ir(piq)_2_(HYNIC)]^+^ to generate hydroxyl radicals (**^•^** OH) and superoxide anion radicals (O_2_ **^•^** ^−^) upon irradiation was investigated using 3′‐(*p*‐hydroxyphenyl) fluorescein (HPF) and dihydrorhodamine 123 (DHR123) as fluorescent probes [27, 28]. An irradiation wavelength of *λ*_exc_ = 420 nm was chosen to match the wavelength used in phototoxicity experiments on cells (vide infra) and was selected to provide access to the MLCT state of each complex whilst avoiding potentially toxic UV irradiation. The *λ*_exc_ = 420 nm light could not be used for the singlet oxygen DPBF assay due to the rapid photobleaching of DPBF when irradiated at this wavelength. Oxidation of HPF by **^•^** OH results in the formation of a fluorescent product [28]. Irradiation (*λ*_exc_ = 420 nm, 26.84 mW/cm^2^) of a mixture of [Ir(piq)_2_(HYNIC)]^+^ and HPF resulted in an increase in fluorescence consistent with the generation of **^•^** OH (Figure S17). In contrast, irradiation of a mixture of Ir(ppy)_2_(HYNIC)]^+^ and HPF did not result in an increase in fluorescence. Oxidation of DHR123 by O_2_ **^•^** ^−^ leads to the formation of fluorescent rhodamine 123 [28]. Irradiation (*λ*_exc_ = 420 nm, 26.84 mW/cm^2^) of a mixture of [Ir(piq)_2_(HYNIC)]^+^ and DHR123 resulted in an increase in fluorescence consistent with the generation of O_2_ **^•^** ^−^ (Figure S18). Irradiation of Ir(ppy)_2_(HYNIC)]^+^ at the same wavelength did not result in the formation of O_2_ **^•^** ^−^ (Figure S18).

The energies of the frontier orbitals in [Ir(ppy)_2_(HYNIC)]^+^ and [Ir(piq)_2_(HYNIC)]^+^ were investigated by cyclic voltammetry. For both complexes, an irreversible process at *E* ^0^ = 0.7 V (vs ferricinium/ferrocene) is attributed to the Ir^IV/III^ process, and one irreversible and two quasi‐reversible processes at lower potentials can be attributed to ligand‐based reductions. Comparison with assignments of the ligand‐based processes in [Ir(ppy)_2_(bpy)]^+^ (bpy = 2,2‐bipyridine) suggests that the first irreversible process, *E* ^0^ = −2.56 V for [Ir(ppy)_2_(HYNIC)]^+^; *E* ^0^ = −2.09 V for [Ir(piq)_2_(HYNIC)]^+^, is due to reduction of the pyridyl ring in the HYNIC ligand (Figure S15). The remaining two quasi‐reversible processes are associated with sequential reductions of the pyridyl groups of the cyclometalating ligands. The reduction waves are more positive (∼40 mV) for [Ir(piq)_2_(HYNIC)]^+^ compared to the [Ir(ppy)_2_(HYNIC)]^+^ and consistent with increased stabilization of the π* orbital of the cyclometalating ligand.

**FIGURE 4:**
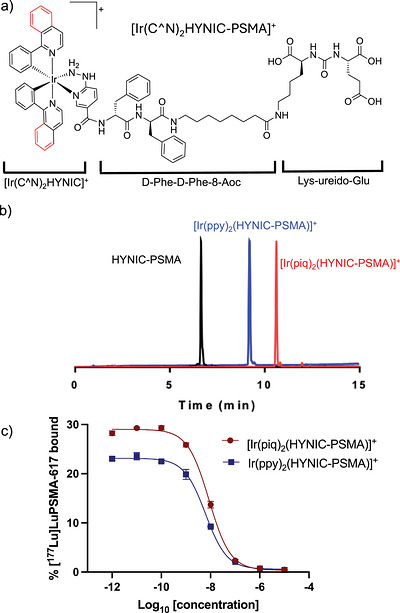
(a) Structure of [Ir(ppy)_2_(HYNIC–PSMA)]^+^ and [Ir(piq)_2_(HYNIC‐PSMA)]^+^ (red). (b) RP‐HPLC traces for HYNIC–PSMA (black), [Ir(ppy)_2_(HYNIC–PSMA)]^+^ (blue), and [Ir(piq)_2_(HYNIC–PSMA)]^+^ (red). (c) Percentage [^177^Lu][LuPSMA‐617] bound versus concentration of either [Ir(ppy)_2_(HYNIC–PSMA)] (blue) or [Ir(piq)_2_(HYNIC–PSMA)] (red). Measurements were performed in triplicate, and error bars are the standard error of the mean.

With the motivation of producing a PSMA‐specific iridium(III) complex, HYNIC was conjugated to a lysine‐ureido‐glutamatic acid pharmacophore through a specifically designed linker consisting of two D‐phenylalanine residues and 8‐aminooctanoic acid (Aoc) to give HYNIC‐D‐Phe‐D‐Phe‐Aoc‐Lys‐ureido‐Glu (HYNIC‐PSMA). HYNIC‐PSMA was synthesized using the standard solid‐phase peptide synthesis. The first step involved immobilization of Fmoc‐Lys(Alloc)─COOH on 2‐chlorotrityl resin. The Fmoc group was removed from the resin‐bound amino acid and reacted with di‐tertbutyl‐L‐glutamate‐isocyanate. Subsequent deprotection and sequential addition of Aoc and two D‐phenylalanine residues were followed by the coupling of HYNIC‐t‐BOC and final deprotection and cleavage of the resin to give HYNIC–PSMA. The iridium complexes, [Ir(ppy)_2_(HYNIC–PSMA)]^+^, and [Ir(piq)_2_(HYNIC–PSMA)]^+^ (Figure 4), were prepared by the reaction of the appropriate iridium(III) precursor with HYNIC–PSMA in a mixture of dichloromethane/methanol and microwave irradiation.

The complexes were purified by semi‐preparative HPLC and characterized by reversed‐phase HPLC and electrospray ionization mass spectrometry with the signals at *m/z* = 1390.5304 Da and *m/z* = 1490.5580 Da for [Ir(ppy)_2_(HYNIC–PSMA)]^+^ and [Ir(piq)_2_(HYNIC–PSMA)]^+^, respectively (Figure 4). The longer HPLC retention time (*R*_t_) of [Ir(piq)_2_(HYNIC–PSMA)]^+^ (*R*_t_ = 10.6 min) when compared to [Ir(ppy)_2_(HYNIC–PSMA)]^+^ (*R*_t_ = 9.2 min) is consistent with the expected increase in lipophilicity. Estimation of the lipophilicity of the conjugates by measuring their octanol to water distribution coefficient (log *D*_7.4_) suggested both complexes are hydrophilic [Ir(ppy)_2_(HYNIC–PSMA)]^+^ (log *D*_7.4_ = −0.2) and [Ir(piq)_2_(HYNIC–PSMA)]^+^ (log *D*_7.4_ = −0.3), which is likely to reduce propensity for cell uptake by passive diffusion.

The ability of both conjugates to bind to LNCaP (human prostate adenocarcinoma) cells that overexpress PSMA enzyme was demonstrated by a competitive binding assay against [^177^Lu][Lu(PSMA‐617)], a complex that is clinically approved for the PSMA‐targeted radionuclide therapy [29, 30]. LNCaP cells were cooled on ice, and [Ir(ppy)_2_(HYNIC–PSMA)]^+^ or [Ir(piq)_2_(HYNIC–PSMA)]^+^ was added at different concentrations (10^−10^–10^−4^ M), followed by the addition of [^177^Lu][Lu(PSMA‐617)]. After 60 min, the amount of radioactive [^177^Lu][Lu(PSMA‐617)] that was bound by the cells was quantified using a gamma‐counter. The nanomolar IC_50_ values of 6.2 × 10^−9^ M for [Ir(ppy)_2_(HYNIC–PSMA)]^+^ and 8.5 × 10^−9^ M for [Ir(piq)_2_(HYNIC–PSMA)]^+^ are consistent with the iridium conjugates having very high affinity for PSMA‐expressing LNCaP cells.

**FIGURE 5:**
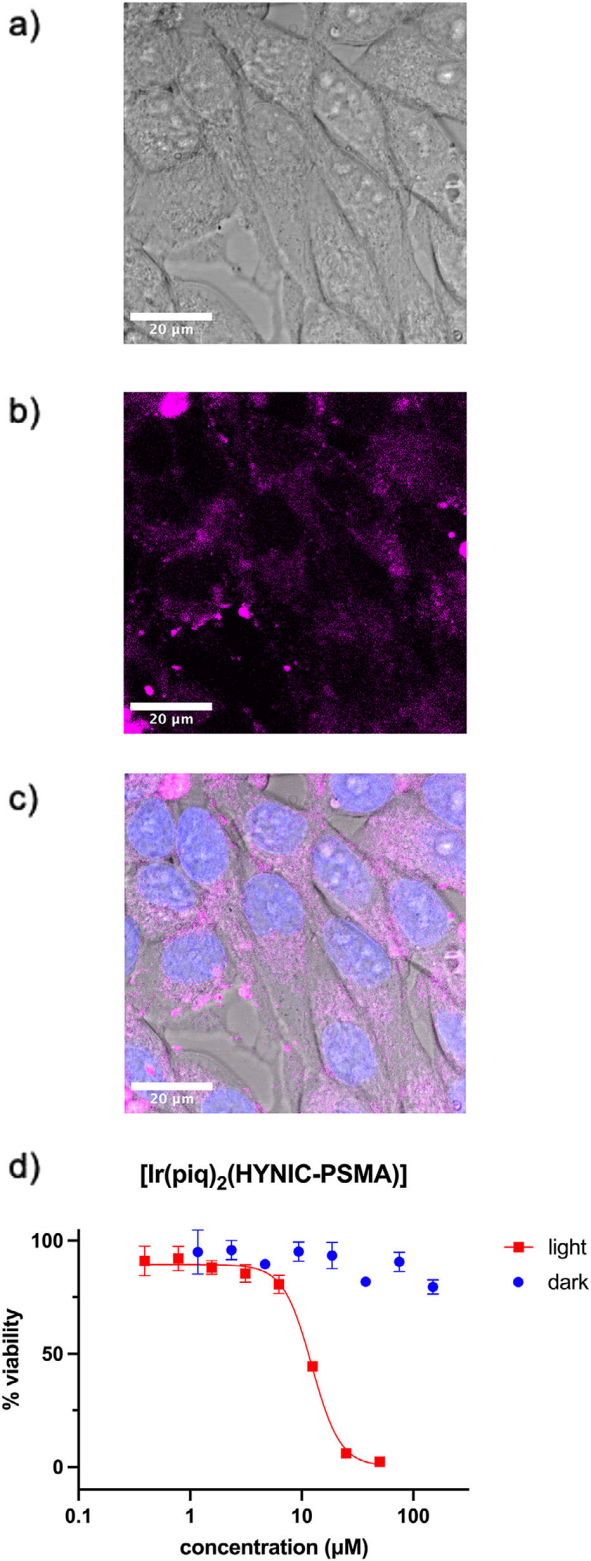
(a–c) Confocal emission microscope image of LNCaP cells treated with [Ir(piq)_2_(HYNIC–PSMA)]^+^ (pink, 25 µM, *λ*_ex_ = 488 nm) and Hoescht 33342 (nuclear stain, blue, *λ*_ex_ = 405 nm). (a) With overlaid brighfield image. (b) *λ*_ex_ = 488 nm channel only. (c) Brightfield image only. (d) Dose‐response curves for [Ir(piq)_2_(HYNIC–PSMA)]^+^in LNCaP cells either in the dark (blue) or with irradiation (red, *λ*_exc_ = 420 nm, 26.84 mW/cm^2^, 30 mins). Measurements performed in triplicate, and error bars are standard error of the mean.

The new conjugates, [Ir(ppy)_2_(HYNIC‐PSMA)]^+^ (*λ*_abs_ = 252, 380 nm; *λ*_em_ = 482, 505 nm (sh)) and [Ir(piq)_2_(HYNIC–PSMA)]^+^ ((*λ*_abs_ = 230, 282, 333, 377, 435 nm; *λ*_em_ = 591 nm), retain the phosphorescence properties of their respective HYNIC complexes. The emission of [Ir(piq)_2_(HYNIC–PSMA)]^+^ can be used to track the cell surface binding and internalization of the complex in PSMA‐positive LNCaP (human prostate adenocarcinoma) cells using confocal emission microscopy (Figure 5). The lower energy of the MLCT for [Ir(piq)_2_(HYNIC–PSMA)]^+^, when compared to [Ir(ppy)_2_(HYNIC–PSMA)]^+^, allows excitation at *λ*_ex_ = 488 nm with minimal autofluorescence (Figure S19). LNCaP cells were treated with the [Ir(piq)_2_(HYNIC–PSMA)]^+^ and Hoechst 33342 (nuclear stain) for 1 h. The media was exchanged, and then the cells were imaged with live cell scanning confocal fluorescence microscopy and transmitted light/bright field imaging. The images are consistent with internalization of [Ir(piq)_2_(HYNIC–PSMA)]^+^, but imaging at later time points and co‐localization studies with endosomal markers would be required to confirm this. PSMA is well known to be an internalizing membrane protein [31], and the images are similar in appearance to examples where PSMA‐binding peptidomimetics have been conjugated to organic fluorophores [32, 33, 34]. These previous studies have demonstrated that PSMA‐binding peptidiomimetics are initially (1 h) internalized by clathrin‐dependent endocytosis, leading to a concentration into endosomes before the peptidomimetic/PSMA enzyme complex dissociates, leaving the peptidomimetic to distribute throughout the cytoplasm whilst the PSMA enzyme recycles to the cell surface [32, 33, 34].

The cytotoxicity and photocytotoxicity of [Ir(piq)_2_(HYNIC–PSMA)]^+^ complexes to LNCaP cells were investigated using standard 3‐(4,5‐dimethylthiazol‐2‐yl)‐2,5‐diphenyltetrazolium bromide (MTT) cell viability assays. Cells were incubated with serial dilutions of [Ir(piq)_2_(HYNIC–PSMA)]^+^ for 1 h, then washed to remove any unbound conjugate. Following a media change, cells were incubated overnight before being incubated with MTT for 2 h. After solubilization of the purple formazan crystals, the absorbance at *λ*_abs_ = 590 nm was measured. [Ir(piq)_2_(HYNIC–PSMA)]^+^ was not cytotoxic at concentrations of at least 150 µM in the dark (Figure 5). Irradiation with light (*λ*_exc_ = 420 nm, 26.84 mW/cm^2^, 30 min) induced dose‐dependent toxicity with an IC_50_ value of 12  µM (Figure 5). In contrast, treatment of cells with [Ir(ppy)_2_(HYNIC‐PSMA)]^+^ and irradiation under the same conditions did not lead to cell death. The lack of phototoxicity of [Ir(ppy)_2_(HYNIC‐PSMA)]^+^ could relate to the fact that the HPF and DHR123 assays suggest that irradiation of [Ir(ppy)_2_(HYNIC)]^+^ with *λ*_exc_ = 420 nm does not result in the formation of either **^•^** OH or O_2_ **^•^** ^−^. This complex does produce singlet oxygen when irradiated at *λ*_exc_ = 365 nm but we were unable to check for singlet oxygen production at *λ*_exc_ = 420 nm using the DPBF probe. Future experiments could investigate different approaches for detecting singlet oxygen following *λ*_exc_ = 420 nm [27].

The potential of [Ir(piq)_2_(HYNIC‐PSMA)]^+^ to elicit phototoxicity by both type I and type II processes could be an advantage when compared to alternative dyes that rely on oxygen‐dependent type II PDT. In principle, the long emission lifetime and large Stokes shift of the iridium complex could allow time‐resolved techniques minimizing background fluorescence and improve contrast for the photodynamic detection of tumor margins. Attaching molecules that bind selectively to enzymes or receptors that are overexpressed in cancer tissue, such as the PSMA enzyme‐targeted here, is another potential advantage when compared to less targeted approaches. Previous examples of molecularly targeted phosphorescent metal complexes include ruthenium and iridium complexes attached to bombesin peptides [35, 36].

A limitation of [Ir(piq)_2_(HYNIC–PSMA)]^+^ is that the blue excitation wavelength used in this work will have limited penetration in tissue. Our preliminary experiments using a mode‐locked titanium:sapphire laser (*λ*_exc_ = 800 nm) demonstrated that two‐photon excitation of [Ir(piq)_2_(HYNIC)]^+^ is possible, which could give better tissue penetration, but more detailed experiments such as measuring the two‐photon absorption cross section are required. It is also acknowledged that the current clinical applicability of two‐photon excitation is limited due to equipment complexity and cost [12].

## 3 Conclusion

New phosphorescent iridium(III) complexes using HYNIC as an auxiliary ligand, a ligand that has been used extensively to form molecularly targeted technetium complexes, have been prepared. The HYNIC ligand has been modified to incorporate a peptidomimetic that binds selectively to the PSMA enzyme. Two conjugates, [Ir(ppy)_2_(HYNIC–PSMA)]^+^ and [Ir(piq)_2_(HYNIC–PSMA)]^+^, were prepared and both the complexes bind to cells that overexpress the PSMA enzyme. The presence of the extended conjugation in the piq ligand in [Ir(piq)_2_(HYNIC‐PSMA)]^+^ results in a bathochromic shift leading to bright red emission (*λ*_em_ = 597 nm). The cell binding and internalization of [Ir(piq)_2_(HYNIC–PSMA)]^+^ were demonstrated in LNCaP cells that overexpress PSMA was demonstrated using confocal emission microscopy. This complex is nontoxic to LNCaP cells in the dark, but irradiation with visible light results in dose‐dependent cytotoxicity. The chemistry reported here could be easily extrapolated to form iridium(III) complexes with other targeting peptides or antibodies. These new complexes have potential applications in identifying tumor margins to guide robot‐assisted surgical resection of tumors as well as molecularly targeted PDT.

## Conflicts of Interest

The authors declare no conflicts of interest

## Supporting information



Full experimental details are included in the Supporting Information. **Supporting File 1**: chem71011‐sup‐0001‐SuppMat.docx.

## Data Availability

The data that support the findings of this study are openly available in the Cambridge Crystallographic Data Centre at https://www.ccdc.cam.ac.uk, reference numbers 2486353, 2486354.

## References

[1] P. A. Sutton , M. A. van Dam , R. A. Cahill , et al., “Fluorescence‐Guided Surgery: Comprehensive Review,” BJS Open 7 (2023): zrad049, 10.1093/bjsopen/zrad049. 37183598 PMC10183714

[2] J. S. D. Mieog , F. B. Achterberg , A. Zlitni , et al., “Fundamentals and Developments in Fluorescence‐Guided Cancer Surgery,” Nature Reviews Clinical Oncology 19 (2022): 9–22, 10.1038/s41571-021-00548-3. 34493858

[3] P. J. Steinkamp , B. K. Pranger , M.‐F. Li , et al., “Fluorescence‐Guided Visualization of Soft‐Tissue Sarcomas by Targeting Vascular Endothelial Growth Factor A: A Phase 1 Single‐Center Clinical Trial,” Journal of Nuclear Medicine 62 (2021): 342–347, 10.2967/jnumed.120.245696. 32680922

[4] M. C. Hekman , M. Rijpkema , C. H. Muselaers , et al., “Tumor‐Targeted Dual‐Modality Imaging to Improve Intraoperative Visualization of Clear Cell Renal Cell Carcinoma: A First in Man Study,” Theranostics 8 (2018): 2161–2170, 10.7150/thno.23335. 29721070 PMC5928878

[5] L. Zhang , X. Shi , Y. Li , et al., “Visualizing Tumors in Real Time: A Highly Sensitive PSMA Probe for NIR‐II Imaging and Intraoperative Tumor Resection,” Journal of Medicinal Chemistry 64 (2021): 7735–7745, 10.1021/acs.jmedchem.1c00444. 34047189

[6] F. W. B. van Leeuwen , T. Buckle , M. N. van Oosterom , and D. D. D. Rietbergen , “The Rise of Molecular Image–Guided Robotic Surgery,” Journal of Nuclear Medicine 65 (2024): 1505–1511, 10.2967/jnumed.124.267783. 38991755

[7] P. Dell'Oglio , D. M. van Willigen , M. N. van Oosterom , et al., “Feasibility of Fluorescence Imaging at Microdosing Using a Hybrid PSMA Tracer During Robot‐Assisted Radical Prostatectomy in a Large Animal Model,” EJNMMI Research 12 (2022): 14, 10.1186/s13550-022-00886-y. 35254544 PMC8901828

[8] Q. Zhou , N. S. van den Berg , W. Kang , et al., “Factors for Differential Outcome Across Cancers in Clinical Molecule‐Targeted Fluorescence Imaging,” Journal of Nuclear Medicine 63 (2022): 1693–1700, 10.2967/jnumed.121.263674. 35332092 PMC9635681

[9] L. C.‐C. Lee and K. K.‐W. Lo , “Luminescent and Photofunctional Transition Metal Complexes: From Molecular Design to Diagnostic and Therapeutic Applications,” Journal of the American Chemical Society 144 (2022): 14420–14440, 10.1021/jacs.2c03437. 35925792

[10] Y. Wu , S. Li , Y. Chen , W. He , and Z. Guo , “Recent Advances in Noble Metal Complex Based Photodynamic Therapy,” Chemical Science 13 (2022): 5085–5106, 10.1039/D1SC05478C. 35655575 PMC9093168

[11] X. Wang , J. Peng , C. Meng , and F. Feng , “Recent Advances for Enhanced Photodynamic Therapy: From New Mechanisms to Innovative Strategies,” Chemical Science 15 (2024): 12234–12257, 10.1039/D3SC07006A. 39118629 PMC11304552

[12] S. Monro , K. L. Colón , H. Yin , et al., “Transition Metal Complexes and Photodynamic Therapy From a Tumor‐Centered Approach: Challenges, Opportunities, and Highlights From the Development of TLD1433,” Chemical Reviews 119 (2019): 797–828, 10.1021/acs.chemrev.8b00211. 30295467 PMC6453754

[13] S. A. McFarland , A. Mandel , R. Dumoulin‐White , and G. Gasser , “Metal‐Based Photosensitizers for Photodynamic Therapy: The Future of Multimodal Oncology?,” Current Opinion in Chemical Biology 56 (2020): 23–27, 10.1016/j.cbpa.2019.10.004. 31759225 PMC7237330

[14] Y. Zhang , B.‐T. Doan , and G. Gasser , “Metal‐Based Photosensitizers as Inducers of Regulated Cell Death Mechanisms,” Chemical Reviews 123 (2023): 10135–10155, 10.1021/acs.chemrev.3c00161. 37534710

[15] M. J. Abrams , M. Juweid , C. I. tenKate , et al., “Technetium‐99m‐Human Polyclonal IgG Radiolabeled via the Hydrazino Nicotinamide Derivative for Imaging Focal Sites of Infection in Rats,” Journal of Nuclear Medicine 31 (1990): 2022–2028. 2266401

[16] D. J. Rose , K. P. Maresca , T. Nicholson , et al., “Synthesis and Characterization of Organohydrazino Complexes of Technetium, Rhenium, and Molybdenum With the {M(η^1^ ‐H_x_ NNR)(η^2^ ‐H_y_ NNR)} Core and Their Relationship to Radiolabeled Organohydrazine‐Derivatized Chemotactic Peptides With Diagnostic Applications,” Inorganic Chemistry 37 (1998): 2701–2716, 10.1021/ic970352f. 11670406

[17] R. C. King , M. B.‐U. Surfraz , S. C. G. Biagini , P. J. Blower , and S. J. Mather , “How Do HYNIC‐Conjugated Peptides Bind Technetium? Insights From LC‐MS and Stability Studies,” Dalton Transactions (2007): 4998, 10.1039/b705111e. 17992285 PMC2258084

[18] L. K. Meszaros , A. Dose , S. C. G. Biagini , and P. J. Blower , “Hydrazinonicotinic Acid (HYNIC)—Coordination Chemistry and Applications in Radiopharmaceutical Chemistry,” Inorganica Chimica Acta 363 (2010): 1059–1069, 10.1016/j.ica.2010.01.009.

[19] N. A. Zia , C. Cullinane , J. K. Van Zuylekom , et al., “A Bivalent Inhibitor of Prostate Specific Membrane Antigen Radiolabeled With Copper‐64 With High Tumor Uptake and Retention,” Angewandte Chemie International Edition 58 (2019): 14991–14994, 10.1002/anie.201908964. 31437347

[20] L. E. McInnes , C. Cullinane , P. D. Roselt , et al., “Therapeutic Efficacy of a Bivalent Inhibitor of Prostate‐Specific Membrane Antigen Labeled With ^67^Cu,” Journal of Nuclear Medicine 62 (2021): 829–832, 10.2967/jnumed.120.251579. 33067341 PMC8729863

[21] K. A. Morgan , S. E. Rudd , A. Noor , and P. S. Donnelly , “Theranostic Nuclear Medicine With Gallium‐68, Lutetium‐177, Copper‐64/67, Actinium‐225, and Lead‐212/203 Radionuclides,” Chemical Reviews 123 (2023): 12004–12035, 10.1021/acs.chemrev.3c00456. 37796539

[22] S. R. Banerjee , K. P. Maresca , K. A. Stephenson , et al., “N , N ‐Bis(2‐mercaptoethyl)Methylamine: A New Coligand for Tc‐99M Labeling of Hydrazinonicotinamide Peptides,” Bioconjugate Chemistry 16 (2005): 885–902, 10.1021/bc050040y. 16029030

[23] S. Sprouse , K. A. King , P. J. Spellane , and R. J. Watts , “Photophysical Effects of Metal‐Carbon .Sigma. Bonds in Ortho‐Metalated Complexes of Iridium(III) and Rhodium(III),” Journal of the American Chemical Society 106 (1984): 6647–6653, 10.1021/ja00334a031.

[24] R. Bevernaegie , S. A. M. Wehlin , B. Elias , and L. Troian‐Gautier , “A Roadmap Towards Visible Light Mediated Electron Transfer Chemistry With Iridium(III) Complexes,” ChemPhotoChem 5 (2021): 217–234, 10.1002/cptc.202000255.

[25] P.‐N. Lai , C. H. Brysacz , M. K. Alam , et al., “Highly Efficient Red‐Emitting Bis‐Cyclometalated Iridium Complexes,” Journal of the American Chemical Society 140 (2018): 10198–10207, 10.1021/jacs.8b04841. 30032607

[26] K. K.‐W. Lo and J. S.‐Y. Lau , “Cyclometalated Iridium(III) Diimine Bis(biotin) Complexes as the First Luminescent Biotin‐Based Cross‐Linkers for Avidin,” Inorganic Chemistry 46 (2007): 700–709, 10.1021/ic0612202. 17257011

[27] D. Abad‐Montero , A. Gandioso , E. Izquierdo‐García , et al., “Ruthenium(II) Polypyridyl Complexes Containing COUBPY Ligands as Potent Photosensitizers for the Efficient Phototherapy of Hypoxic Tumors,” Journal of the American Chemical Society 147 (2025): 7360–7376, 10.1021/jacs.4c15036. 39953993 PMC12164272

[28] M. Li , J. Xiong , Y. Zhang , et al., “New Guidelines and Definitions for Type I Photodynamic Therapy,” Chemical Society Reviews 54 (2025): 7025–7057, 10.1039/D1CS01079D. 40539837

[29] M. Benešová , M. Schäfer , U. Bauder‐Wüst , et al., “Preclinical Evaluation of a Tailor‐Made DOTA‐Conjugated PSMA Inhibitor With Optimized Linker Moiety for Imaging and Endoradiotherapy of Prostate Cancer,” Journal of Nuclear Medicine 56 (2015): 914–920. 25883127 10.2967/jnumed.114.147413

[30] U. Hennrich and M. Eder , “[177Lu]Lu‐PSMA‐617 (PluvictoTM): the First FDA‐Approved Radiotherapeutical for Treatment of Prostate Cancer,” Pharmaceuticals 15 (2022): 1292, 10.3390/ph15101292. 36297404 PMC9608311

[31] S. A. Rajasekaran , G. Anilkumar , E. Oshima , et al., “A Novel Cytoplasmic Tail MXXXL Motif Mediates the Internalization of Prostate‐Specific Membrane Antigen,” Molecular Biology of the Cell 14 (2003): 4835–4845, 10.1091/mbc.e02-11-0731. 14528023 PMC284788

[32] G. G. Simpson , J. M. Quintana , J. E. Carrothers , et al., “Fluorescent PSMA‐Targeted Radiotheranostic Compounds for Multiscale Imaging,” Bioconjugate Chemistry 36 (2025): 1448–1460, 10.1021/acs.bioconjchem.5c00139. 40574660 PMC12365983

[33] S. A. Kularatne , K. Wang , H.‐K. R. Santhapuram , and P. S. Low , “Prostate‐Specific Membrane Antigen Targeted Imaging and Therapy of Prostate Cancer Using a PSMA Inhibitor as a Homing Ligand,” Molecular Pharmaceutics 6 (2009): 780–789, 10.1021/mp900069d. 19361233

[34] J. Matthias , J. Engelhardt , M. Schäfer , et al., “Cytoplasmic Localization of Prostate‐Specific Membrane Antigen Inhibitors May Confer Advantages for Targeted Cancer Therapies,” Cancer Research 81 (2021): 2234–2245, 10.1158/0008-5472.CAN-20-1624. 33622696

[35] M. J. S. A. Silva , R. Vinck , Y. Wang , et al., “Towards Selective Delivery of a Ruthenium(II) Polypyridyl Complex‐Containing Bombesin Conjugate Into Cancer Cells,” Chembiochem 24 (2023): e202200647, 10.1002/cbic.202200647. 36479913

[36] J. Sanz‐Villafruela , C. Bermejo‐Casadesús , G. Riesco‐Llach , et al., “Bombesin‐Targeted Delivery of β‐Carboline‐Based Ir(III) and Ru(II) Photosensitizers for a Selective Photodynamic Therapy of Prostate Cancer,” Inorganic Chemistry 63 (2024): 19140–19155, 10.1021/acs.inorgchem.4c02583. 39361042 PMC11483813

