# Supplementary material for: Phosphorescent Iridium Hydrazinonicotinic Acid (HYNIC) Complexes That Bind to Prostate Specific Membrane Antigen: Potential Photodynamic Therapy of Prostate Cancer
Source: Chemistry. 2026 Apr 17;32(25):e71011. doi: 10.1002/chem.71011 (PMC13331583; doi:10.1002/chem.71011)
Supplement: Supplementary file 1 — Full experimental details are included in the Supporting Information. Supporting File 1: chem71011‐sup‐0001‐SuppMat.docx. [file CHEM-32-e71011-s001.docx]

**Phosphorescent Iridium Hydrazinonicotinic (HYNIC) Complexes that Bind to Prostate Specific Membrane Antigen: Potential Photodynamic Detection and Photodynamic Therapy of Prostate Cancer**

La’El Kimchi, Emily R. McGowan, Jonathan M. White, Trevor A. Smith, Stacey E. Rudd, and Paul S. Donnelly*

School of Chemistry and Bio21 Molecular Science and Biotechnology Institute, University of Melbourne, Melbourne, Victoria, Australia 3010

*corresponding author: pauld@unimelb.edu.au

**Supporting Information**

**Table of contents:**

Experimental

1. General

2. Synthesis

3. X-ray crystallography

4. Electronic Spectroscopy and Electrochemistry

5. Investigation of reactive oxygen species generation

6. LogP determination for HYNIC-PSMA complexes

7. Cell culture

8. Live cell confocal microscopy

9. Cytotoxicity and phototoxicity assays

10. Competitive binding assay

**Experimental**

**1. General**

All reagents and solvents were purchased from commercial suppliers and were used as supplied. NMR spectra were recorded on either a JEOL (JNM-ECZ400R) spectrometer (^1^H at 400 MHz) or Bruker NEO spectrometer equipped with a QR cryoprobe (^1^H at 500 Mz and ^13^C at 125.8). ^19^F NMR spectra were acquired 76 MHz on a Magritek Benchtop Spinsolve spectrometer. Chemical shifts are expressed in parts per million (ppm) and were referenced to solvent peaks. Electrospray Ionisation Mass Spectra (ESI-MS) were acquired with a Thermo Orbitrap Mass Exactive mass spectrometer set in the positive ion mode. Analytical Reversed Phase High Performance Liquid Chromatography (RP-HPLC) chromatograms were acquired on Agilent 1100 series equipped with a Phenomenex C_18_ LUNA Column (150 x 4.6 mm) (Phenomenex, CA) with detection at λ_abs_ 254 nm and λ_abs_ 280 nm. Semi-Preparative RP-HPLC was conducted on an Agilent 1260 Infinity II Preparative LC System equipped with an Agilent Pursuit XR column (21 mm x 250 mm, 5 μm) and various gradient elutions of acetonitrile in water with 0.1% formic acid at a flow rate of 12.5 mL/min, with detection at λ = 254 nm and λ = 280 nm.

Absorbance spectra were recorded on a UV-1650 PC spectrophotometer (Shimadzu, Kyoto, Japan). Samples were measured at concentrations between 5-25 µM, and the extinction coefficients calculated using the Beer-Lambert law. Emission spectra were recorded on a Cary Eclipse spectrophotometer (Varian, CA, USA) with excitation bandwidth set at 5 nm and an emission bandwidth set at 10 nm. Absorption and emission spectra were recorded in aerated acetonitrile. Solutions prepared had an absorbance of <= 0.1 at the excitation wavelength, and irradiated at λ_exc_ = 380, 440 ~~nm~~. Luminescence quantum yield were conducted in both acetonitrile and deoxygenated phosphate buffered saline (PBS) (99:1 PBS/DMSO (v/v), pH = 7.4). Solutions for these measurements were prepared as described. Quantum yields were measured relative to known standard [Ru(bpy)_3_]Cl_2_.^[1]^ Time-resolved luminescence (lifetime) measurements were carried out on dilute solutions in deaerated CH_3_CN using a tunable Q-switched Nd:YAG laser/optical parametric oscillator system (EKSPLA NT342), operating at 10 Hz with λ_exc_= 380 nm or 440 nm. Emission was monitored at selected wavelengths using a spectrograph (Acton Research Corporation SpectraPro 300i) with a 150 g/mm 500 nm blazed grating and detected with a photomultiplier (Hamamatsu R928, ~ 15 ns response) fed to a digital oscilloscope (Teledyne LeCroy WaveSurfer 10). The decay curves fitted by a simple exponential decay function to extract the emission decay time.

Cyclic voltammetry measurements were performed using an EDAQ potentiostat 466 workstation (Model ER466) and data processed with EDAQ ECHEM V2.2.3 software. Measurements were conducted using a glassy carbon working electrode, a platinum wire counter electrode and a leakless Ag/Ag^+^ reference electrode. Measurements were carried out in degassed dimethylformamide that had been dried over 3 Å molecular sieves prior to use. Tetrabutylammonium hexafluorophosphate (TBA-PF­_6_) was used as the supporting electrolyte. Measurements performed under an argon atmosphere, and redox potentials referenced to the ferrocene-ferricenium redox couple (E°’(Fc/Fc^+^) = 0.00).

All microwave reactions were conducted using a Biotage Initiator(+) workstation (Upsalla, Sweden) using the supplied reaction vials.

**2. Synthesis**

[Ir(ppy)_2_(µ-Cl]_2_ and [Ir(piq)_2_(µ-Cl]_2_,^[2]^ and 6-Hydrazinopyridine-3-carboxylic acid (HYNIC) and 6-*t*Boc-hydrazinopyridine-3-carboxylic acid (HYNIC-BOC) were prepared as described previously.^[3]^

***2.1 Synthesis of [Ir(ppy)_2_HYNIC]PF_6_, bis[2-phenylpyridinato-N,C^2’^][6-hydazinonicotinicacid-N,N’]iridium(III) hexafluorophosphate***

[Ir(ppy)_2_(*µ*-Cl]_2_ (0.160 g, 0.149 mmol) and 6-hydrazinonicotinc acid (0.0592 g, 0.386 mmol) were added to a deoxygenated mixture of dichloromethane/methanol (3:1). The mixture was heated at reflux for 4 hours under an atmosphere of dinitrogen. The mixture was then allowed to cool to room temperature and then filtered. The filtrate was evaporated to dryness under reduced pressure and the residue was re-dissolved in ethanol and filtered again. This filtrate was evaporated to dryness under reduced pressure and the residue was dried *in vacuo*. To this residue was added water (10 mL) and the mixture was acidified to pH ~ 1 with HPF_6_ (1 M) which resulted in precipitation of a dark green powder. This precipitate was collected by filtration and washed with copious amounts of water, ice-cold ethanol and diethyl ether to give [Ir(ppy)_2_HYNIC]PF_6_ as a dark green powder (0.144 g (0.180 mmol, 72%). HR-ESIMS (+ ion) [C_28_H_23_IrN_5_O_2_]^+^ *m/z* = 654.1482 (experimental), 654.1476 (calculated); ^1^H-NMR (500 MHz; DMSO-d_6_): δ 10.57 (s, 1H), 8.90 (d, *J* = 5.7 Hz, 1H), 8.52 (d, *J* = 9.8 Hz, 1H), 8.25^1^H (d, *J* = 8.2 Hz, 1H), 8.21 (d, *J* = 8.2 Hz, 1H), 7.96 (ddt, *J* = 24.9, 16.6, 7.8 Hz, 5H), 7.81 (t, *J* = 7.8 Hz, 2H), 7.66 (s, 1H), 7.38 (t, *J* = 6.5 Hz, 1H), 7.34 (t, *J* = 6.5 Hz, 1H), 6.92 (t, *J* = 7.4 Hz, 2H), 6.87 (t, *J* = 7.4 Hz, 1H), 6.79 (t, *J* = 7.4 Hz, 1H), 6.73 (t, *J* = 7.4 Hz, 1H), 6.22 (d, *J* = 7.5 Hz, 1H), 6.11 (d, *J* = 7.5 Hz, 1H); {^1^H}^13^C NMR (126 MHz; DMSO-d_6_) 27 resonances for 28 carbon atoms suggests two isochronous carbon nuclei: δ 168.0, 167.4, 165.3, 161.5, 152.2, 150.0, 148.9, 148.3, 147.6, 144.8, 143.9, 138.6, 138.5, 138.4, 132.4, 130.9, 130.0, 129.1, 124.9, 124.4, 123.6, 123.3, 121.6, 121.4, 119.9, 119.7, 106.8; ^19^F-NMR (76 MHz; DMSO-d_6_): δ -73.79 (d, J = 711.2 Hz, 6F); Elemental analysis calculated for IrC_28_H_23_N_5_O_2_⦁PF_6_ ; C 42.11% H 2.90% N 8.28 %; found C 45.54 % H 3.17% N 8.18 %; UV-Vis (CH_3_CN) λ_max_: 255 nm (42020 M^-1^ cm^-1^) 383 nm (4060 M^-1^ cm^-1^) Emission (CH_3_CN, λ_exc_ 380 nm) 485 nm; Φ 0.26 (CH_3_CN) 0.31 (99:1 PBS/DMSO (v/v), pH = 7.4)), τ 2.58 µs (CH_3_CN).

Crystals suitable for single crystal X-ray crystallography were grown from a solution of the complex dissolved in methanol.


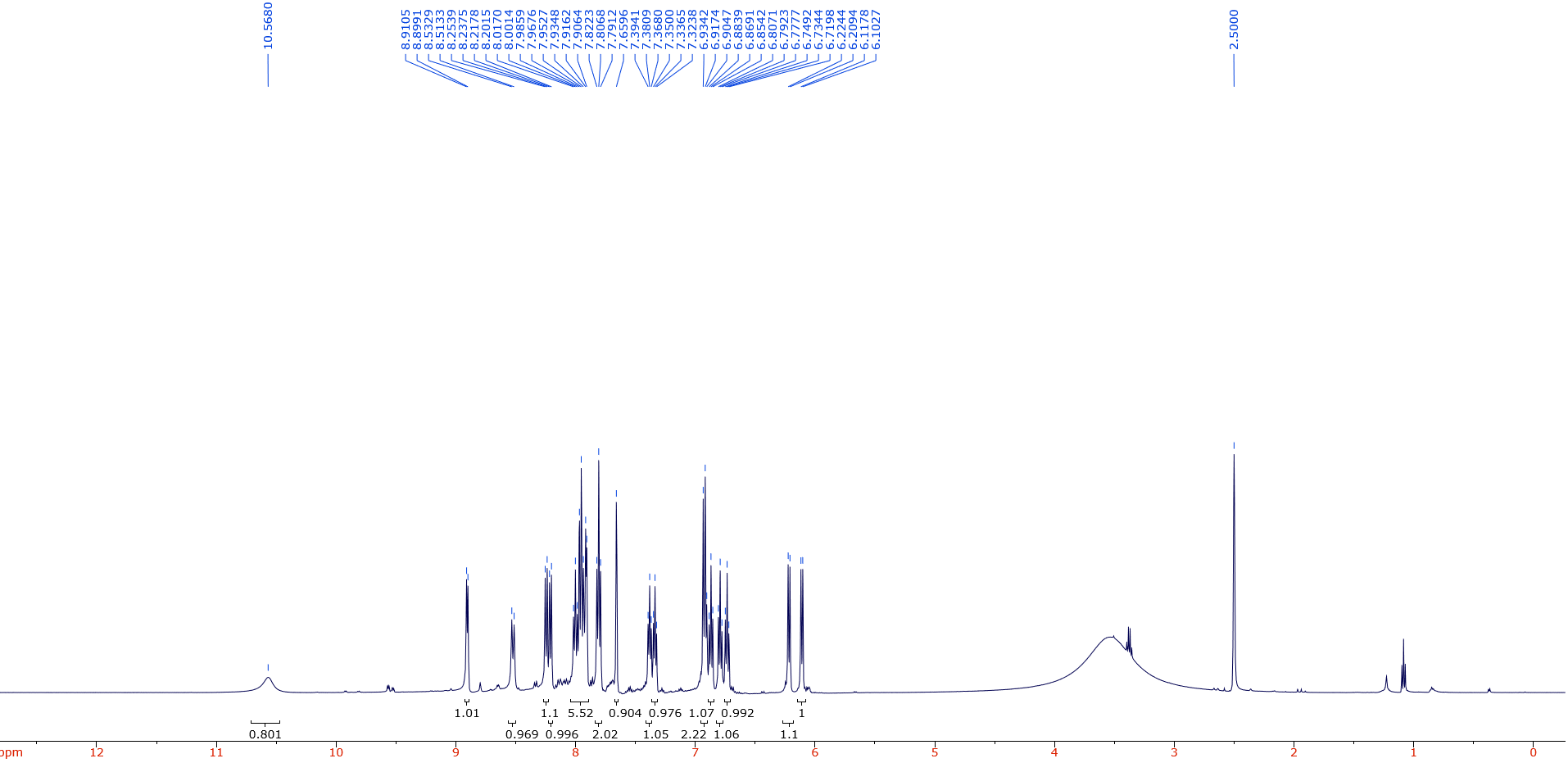


**Fig. S1.** ^1^H NMR spectrum (500 MHz) of [Ir(ppy)_2_(HYNIC]PF_6_ in d_6_-DMSO.

**
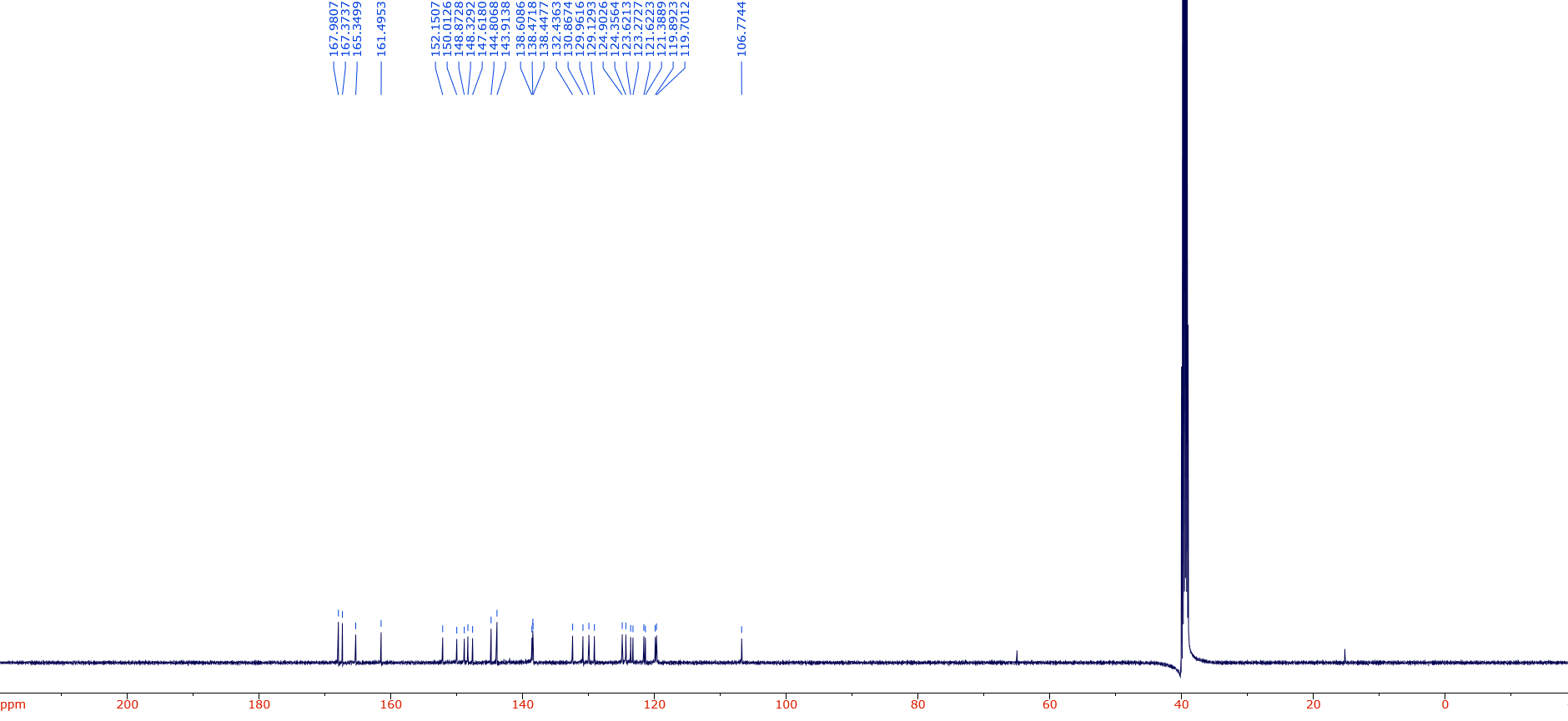
**

**Fig. S2.** Fig {^1^H}^13^C NMR spectrum (125.8 Mz) of [Ir(ppy)_2_(HYNIC]PF_6_ in d_6_-DMSO.

**
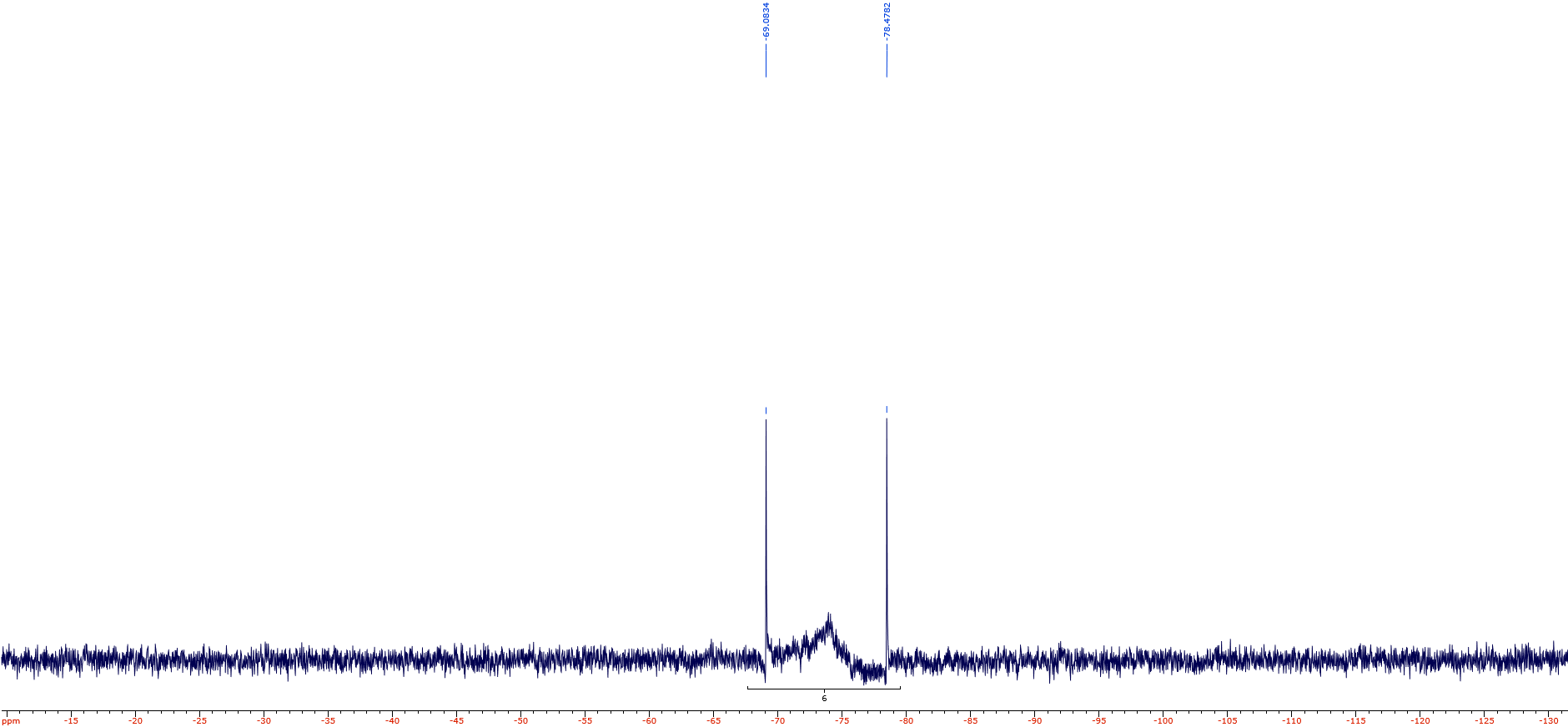
**

Fig. S3. ^19^F NMR spectrum (75 MHz) of [Ir(ppy)_2_(HYNIC)]PF_6_ in d^6^-DMSO.

**Fig. S4.** ^1^H NMR analysis of [Ir(ppy)_2_(HYNIC)]^+^ in d_6_-DMSO over 24 hours under exposure to ambient laboratory conditions suggests the complex is stable under these conditions.

**Fig. S5.** ESI-MS spectrum positive ion mode for [Ir(ppy)_2_(HYNIC)]PF_6_.

***2.2 Synthesis of [Ir(piq)_2_HYNIC][PF_6_], bis[2-phenylisoquinolinato-N,C^2’^][6-hydazinonicotinic acid-N,N’]iridium(III) hexafluorophosphate***

[Ir(piq)_2_-(µ-Cl)]_2_ (0.053 g, 0.042 mmol) and 6-hydrazinonicotinic acid (0.016 g, 0.11 mmol were added to a deoxygenated mixture of dichloromethane/methanol (3:1). The mixture was heated at reflux for 4 hours under an atmosphere of dinitrogen. The mixture was then allowed to cool to room temperature and then filtered. The filtrate was evaporated to dryness under reduced pressure and the residue was re-dissolved in ethanol and filtered again. This filtrate was evaporated to dryness under reduced pressure and the residue was dried *in vacuo*. To this residue was added water (10 mL) and the mixture was acidified to pH ~ 1 with HPF_6_ (1 M) which resulted in precipitation of a dark red powder. This precipitate was collected by filtration and washed with copious amounts of water, ice-cold ethanol and diethyl ether to give [Ir(piq)_2_HYNIC][PF_6_] as a dark red powder (0.040 g, 0.045 mmol, 53%). HR-ESIMS (+ ion) [C_36_H_27_IrN_5_O_2_]^+^ *m/z* = 754.1787 (experimental) 754.1789 (calculated); ^1^H-NMR (500 MHz; DMSO-d_6_): δ 10.55 (s, 1H), 9.00 (d, *J* = 8.3 Hz, 1H), 8.94 (d, *J* = 8.5 Hz, 1H), 8.72 (d, *J* = 6.5 Hz, 1H), 8.53 (d, *J* = 10.1 Hz, 1H), 8.27 (t, *J* = 8.1 Hz, 2H), 8.17-8.14 (m, 2H), 7.97-7.83 (m, 9H), 7.79 (d, *J* = 6.5 Hz, 1H), 7.56 (d, *J* = 1.8 Hz, 1H), 7.05-7.02 (m, 1H), 7.00-6.94 (m, 2H), 6.82-6.79 (m, 1H), 6.75-6.72 (m, 1H), 6.24 (dd, *J* = 7.6, 0.9 Hz, 1H), 6.18 (dd, *J* = 7.7, 0.8 Hz, 1H) ; ^13^C-NMR (126 MHz; DMSO-d_6_) 35 resonances for 36 carbon atoms suggests two isochronous carbon nuclei: δ 168.8, 168.2, 165.1, 161.5, 155.6, 151.2, 148.2, 146.0, 145.1, 141.8, 140.8, 138.6, 136.8, 136.6, 132.9, 131.9, 131.7, 131.4, 130.5, 130.2, 129.8, 129.3, 129.2, 129.0, 127.7, 127.6, 126.5, 126.4, 126.0, 125.4, 122.0, 121.6, 121.5, 121.3, 106.7.^19^F-NMR (76 MHz; DMSO-d_6_): δ -73.79; (d, *J* = 711.3 Hz, 6F); Elemental analysis calculated for IrC_36_H_27_N_5_O_2_⦁PF_6_ ; C 48.11% H 3.03% N 7.79% Found C 51.22% H 3.31%;N 7.07% UV-Vis (CH_3_CN) λ_max_: 234 nm (47840 M^-1^ cm^-1^) 289 nm (33600 M^-1^ cm^-1^) 335(sh) nm (15860 M^-1^ cm^-1^) 387(sh) nm (7500 M^-1^ cm^-1^) 435 nm (5620 M^-1^ cm^-1^) Emission (CH_3_CN, λ_exc_ 440 nm) 598 nm; Φ 0.20 (MeCN) 0.009 (99:1 PBS/DMSO (v/v), pH = 7.4)), τ 2.26 µs (CH_3_CN)

Crystals suitable for single crystal X-ray crystallography were grown by adding a few drops of concentrated hydrochloric acid to solution of the complex dissolved in acetonitrile. Exchange of vapours between this solution and diethyl ether allowed isolation of dark red crystals of [Ir(piq)_2_HYNIC]Cl•CH_3_CN•(CH_3_CH_2_)O.


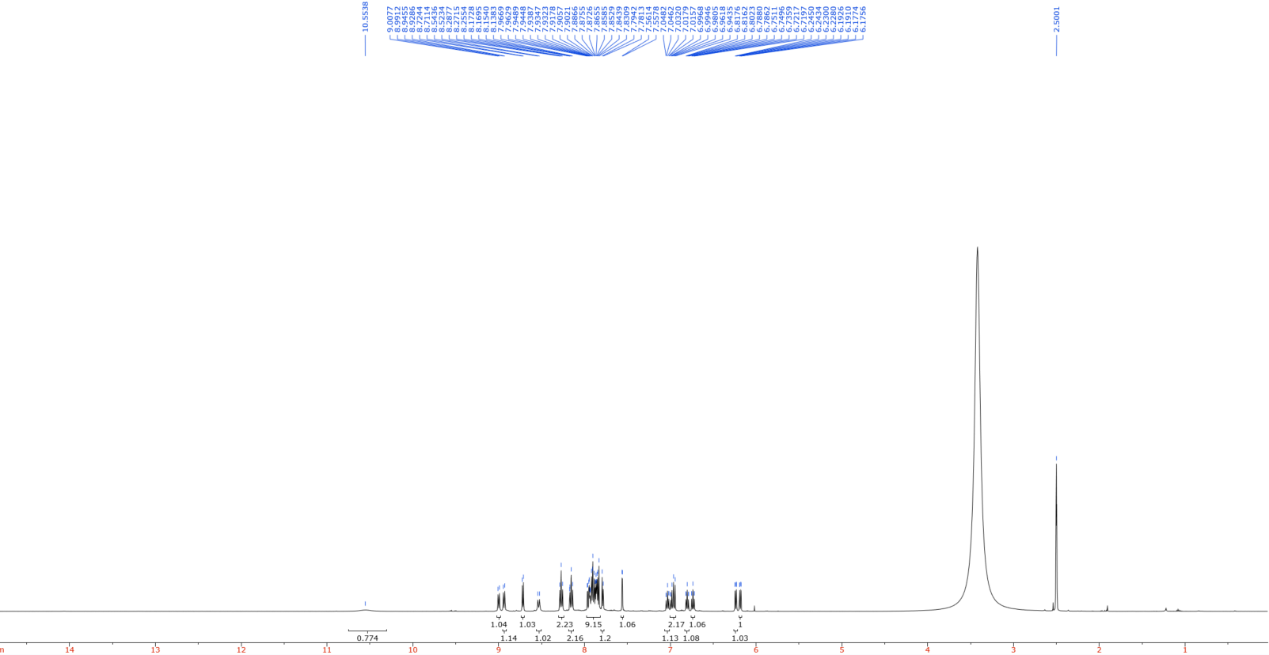


**Fig. S6.** ^1^HNMR spectrum (500 MHz) of [Ir(piq)_2_(HYNIC]PF_6_ in d_6_-DMSO.


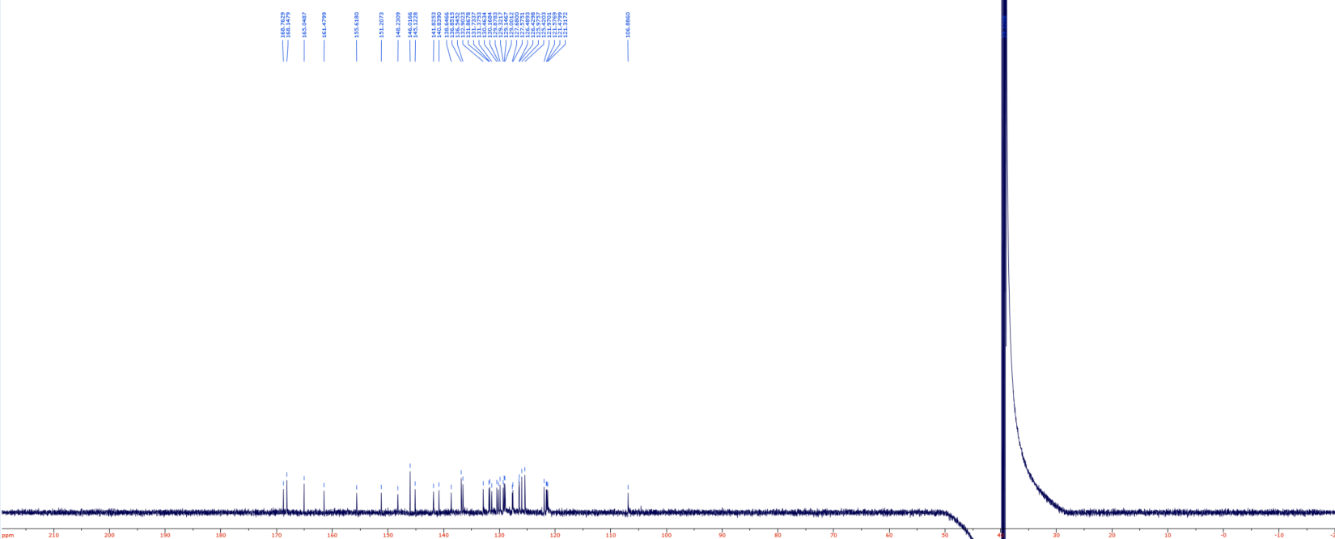


**Fig. S7.** {^1^H}^13^C NMR spectrum (125.8 Mz) of [Ir(piq)_2_(HYNIC]PF_6_ in d_6_-DMSO.


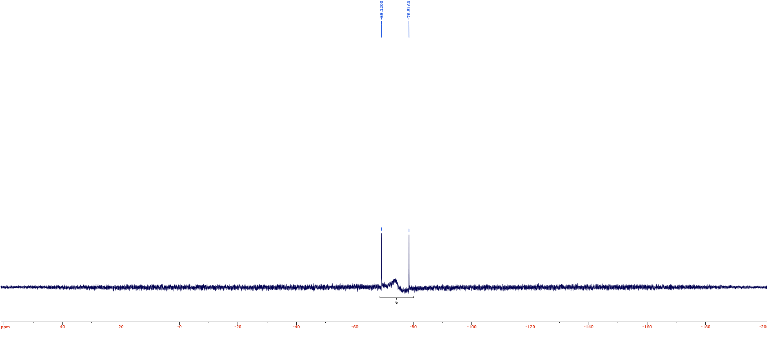


**Fig. S8.** ^19^F NMR spectrum (75 MHz) of [Ir(piq)_2_(HYNIC)]PF_6_ in d^6^-DMSO.

**Fig. S9.** ESI-MS spectrum positive ion mode for [Ir(piq)_2_(HYNIC)]PF_6_.

***2.3 Synthesis of HYNIC-PSMA (12)***

See Scheme S1 for compound numbering. Compounds 1-9 were prepared by modifications of reported procedures.^[4]^

- A) Fmoc-Lys(Alloc)-COOH (**1**) (2.2 g, 5 mmol), 1-hydroxybenzotriazole (675 mg, 5 mmol), 4-dimethylaminopyridine (0.2 mmol, 25 mg) and N,N’-diisopropylcarbodiimide (630 mg, 770 μL, 5 mmol) were added to a suspension of Wang resin (1.8 g, 2 mmol) in 9:1 dichloromethane/dimethylformamide (20 mL) and rocked at room temperature for 4 h. The resin beads were isolated by filtration and washed with dichloromethane and diethyl ether. The resin was resuspended in 20 mL 17:2:1 dichloromethane/acetic anhydride/pyridine and rocked at room temperature for a further 30 minutes. The resin beads were isolated by filtration and washed with dichloromethane and diethyl ether. The resin was resuspended in 20% piperidine in dimethylformamide (20 mL) and rocked at room temperature for 30 minutes to remove the Fmoc protecting group. The resin beads were isolated by filtration and washed with dimethylformamide, dichloromethane and diethyl ether to give **2**.
- B) Carbonyl diimidazole (2.1 g, 13.2 mmol) was dissolved in dichloromethane (20 mL) at 0 ºC. A solution of di-tertbutyl-L-glutamate hydrochloride (**3**) (3.5 g, 12 mmol) in dichloromethane (25 mL) and diisopropylethylamine (3 mL) was added to the carbonyl diimidazole solution dropwise and the reaction mixture was stirred at 0 ºC for 3 h to give **4** in situ.
- C) The resin beads from **2** were added directly to this mixture which was allowed to warm to room temperature and stirred gently for 18 h. The resin beads were isolated by filtration and washed with dichloromethane and diethyl ether. A second iteration of the coupling was performed in the same manner to give **5**.
- D) **5** was then suspended in dichloromethane (20 mL), and to this a mixture of [Pd(PPh_3_)_4_]­ (460 mg, 0.4 mmol) and morpholine (900 µL, 10 mmol) was added and the suspension rocked at room temperature for 3 hours. The resin beads were washed with dichloromethane and dried with diethyl ether to give compound **6**.
- E) Fmoc-8-aminoctanoic acid (2.2 g, 6 mmol), HATU (2.2 g, 6 mmol) and diisopropylethylamine (1 mL) were dissolved in dimethylformamide (20 mL) and added to preswelled resin beads of compound **6** and microwave irradiated at 50 °C for 30 minutes. The terminal fmoc protecting group was removed as described in step **a** to give compound **7**.
- G) Fmoc-D-phenylalanine (2.3 g, 6 mmol), HATU (2.2 g, 6 mmol) and diisopropylethylamine (1 mL) were dissolved in dimethylformamide (20 mL) and added to preswelled resin beads of compound **7** and microwave irradiated at 50 °C for 30 minutes. The terminal fmoc protecting group was removed as described in step **a**. A second D-phenylalanine residue was installed using the same procedure followed by a final fmoc deprotection to afford compound **8.**
- H) Boc-HYNIC **(9)** (0.38 g, 1.5 mmol), HATU (0.57 g, 1.5 mmol) and diisopropylethylamine (250 μL) were dissolved in dimethylformamide (10 mL) and added to one quarter of the pre-swelled resin beads of compound **8** and microwave irradiated at 50 °C for 30 minutes. The resin beads were isolated by filtration, and washed with dimethylformamide, dichloromethane and diethyl ether to afford compound **10.**
- I) Compound **10** was cleaved from the solid support by suspending in a trifluoroacetic acid/triisopropylsilane/H_2_O (90%/5%/5%) (10 mL) mixture and rocking at room temperature for 1.5 hours. The resin beads were removed by filtration, and the filtrate reduced under a stream of N_2_. Addition of ice-cold diethyl ether followed by centrifugation elicited the precipitation of an off-white solid. The supernatant was decanted, and the residue dissolved in a 30% acetonitrile in H_2_O mixture and lyophilised. The crude peptide was then purified using semi-preparative HPLC (30-40% acetonitrile in water with 0.1% formic acid, over 20 minutes) to afford compound **12** (HYNIC-PSMA), as a white fluffy powder (36 mg, 8%). ESI(+)-HRMS [C_44_H_59_N_9_O_11_+H]^+^ m/z 890.4407 (experimental) 890.4508, [C_44_H_59_N_9_O_11_+2H]^2+^ m/z 445.7240 (experimental) 445.7295. Rt = 6.7 min (10-90 % B in A (acetonitrile in H_2_O, 0.1% trifluoracetic acid (TFA) over 15 minutes), RT = 9.2 (10-95 % B in A (acetonitrile in H_2_O, 0.1% TFA) over 25 minutes).

**Scheme S1.** a) i) Wang resin, dichloromethane, diisopropylethylamine. ii) 20% piperidine, dimethylformamide; b) carbonyl diimide, dichloromethane, diisopropylethylamine; c) RT stirring; d) [Pd(PPh_3_)_4_]­ (10 mmol %), morpholine, dichloromethane; e) Fmoc-8-aminooctanoic acid, HATU, diisopropylethylamine, dimethylformamide; f) Fmoc-D-Phenylalanine, HATU, diisopropylethylamine, dimethylformamide; repeated twice; g) HATU, diisopropylethylamine, dimethylformamide; h) boc-HYNIC, HATU, dimethylformamide, diisopropylethylamine i) trifluoroacetic acid/triisopropylsilane/H_2_O (90%/5%/5%).

**Fig. S10.** Characterisation of HYNIC-PSMA. a) HPLC chromatogram of HYNIC-PSMA (mobile phase gradient 10-95% B in A (acetonitrile in H_2_O with 0.1% TFA) over 15 minutes (detection at λ = 254 nm). b) HPLC chromatogram of HYNIC-PSMA (mobile phase gradient 10-95% B in A (acetonitrile in H_2_O with 0.1% TFA) over 25 minutes (detection at λ = 254 nm). c) ESI-MS of HYNIC-PSMA (positive ion mode).

**2.4 *Synthesis of [Ir(ppy)_2_(HYNIC-PSMA)]^+^***

[Ir(ppy)_2_(µ-Cl)]_2_ (7.5 mg, 0.007 mmol) and HYNIC-PSMA (10 mg, 0.012 mmol) were combined in 3:1 dichloromethane/methanol (5 mL) and the mixture was irradiated in a microwave reactor at 50 °C for 1 h. The solvent was removed under reduced pressure and the residue dissolved in 30:70 acetonitrile/water and lyophilized. Purification by semi preparative HPLC (30-70% over 20 minutes, acetonitrile in H_2_O, 0.1% formic acid). Lyophilisation of the appropriate fractions allowed isolation of a light-yellow powder (8.1 mg, 49%, estimated purity from HPLC ~95%). ESI(+)-HRMS [C_66_H_75_N_11_O_11_Ir]^+^ m/z (calculated) 1390.5272, (experimental) 1390.5298, [C_66_H_75_N_11_O_11_Ir+H]^2+^ m/z (calculated) 695.7672, (experimental) 695.7676. HPLC; RT = 9.1925 min (10-95% over 15 minutes acetonitrile in H_2_O, 0.1% TFA).

**^^**

**Fig S11**. a) HPLC chromatogram of [Ir(ppy)_2_(HYNIC-PSMA)]^+^. b) ESI-MS of [Ir(ppy)_2_(HYNIC-PSMA)]^+^ c) Absorbance and fluorescence spectra of [Ir(ppy)­_2_(HYNIC-PSMA)]^+^ in 70/30 acetonitrile/water. Concentration estimated to be 12.5 µM based on the extinction coefficient of the MLCT ( λ_max_ 383 nm).

**2.5 *Synthesis of [Ir(piq)_2_(HYNIC-PSMA)]^+^***

[Ir(piq)_2_-(µ-Cl)]_2_ (14 mg, 0.0108 mmol) and HYNIC-PSMA (15 mg, 0.018 mmol) were combined in a mixture 3:1 dichloromethane/methanol (5 mL). This mixture was heated by microwave irradiation at 50 °C for 1 h. The solvent was then removed under reduced pressure. The residue was dissolved in acetonitrile/water (40% v/v) and then lyophilised. Purification by semi preparative HPLC (40-70% over 20 minutes acetonitrile in H_2_O, 0.1% formic acid), allowed isolation of a fluffy red powder (15 mg, 56 %, estimated purity from HPLC ~95%). ESI(+)-HRMS [C_74_H_79_N_11_O_11_Ir]^+^ m/z (calculated) 1490.5590, (experimental) 1490.5598, [C_74_H_79_N_11_O_11_Ir +H]^2+^ m/z (calculated) 745.7829, (experimental) 745.7824, RT = 10.6067 minutes (10-95 % MeCN in H_2_O, 0.1% TFA) over 15 minutes.

**Fig S12**. a) HPLC chromatogram of [Ir(piq)_2_(HYNIC-PSMA)]^+^. b) ESI-MS of [Ir(piq)_2_(HYNIC-PSMA)]^+^ c) Absorbance and fluorescence spectrum of [Ir(piq)_2_(HYNIC-PSMA)]^+^ in a 50/50 acetonitrile/water. Concentration estimated to be 14.5 µM from the extinction coefficient of the MLCT ( λ_max_ 440 nm).

**3. X-ray Crystallography**

Crystals were mounted in low temperature oil and cooled to 100 K. Intensity data was collected using a XtaLAB Synergy Rigaku Oxford X-ray diffractometer using either Mo Kα (λ = 0.71073 Å) or Cu Kα (λ = 1.54184 Å) radiation (Table S1). The structures were solved by direct methods and Fourier analysis using SHELX suite of programs,^[5]^ as implemented within the OLEX software package.^[6]^ Thermal ellipsoids were generated using the Mercury software program.^[7]^ CDCC deposition numbers 2486354 (for [Ir(ppy)(HYNIC)]) and 2486353 (for [Ir(piq)_2_(HYNIC)]Cl.CH_3_CN.(CH_3_CH_2_)_2_O).

| **Name** | **[Ir(ppy)_2_HYNIC]** | **[Ir(piq)_2_HYNIC]Cl**  **•CH_3_CN•(CH_3_CH_2_)O** |
| --- | --- | --- |
| Chemical formula | C_28_H_22_IrN_5_O_2_ | C_82_H_77_Cl_2_Ir_2_N_11_O_6_ |
| *M_w_* | 652.70 | 1767.84 |
| T/K | 100.00(10) K | 100.00(10) |
| Wavelength/Å | 0.71073 | 1.54184 |
| Crystal System | Monoclinic | triclinic |
| Space group | P 21/c | P-1 |
| *a* / Å | 12.1916(3) | 14.5512(4) |
| *b* / Å | 14.4976(5) | 16.4499(6) |
| *c* / Å | 17.3879(5) | 17.5997(5) |
| *α* / ° | 90 | 67.382(3) |
| *β* / ° | 98.672(2) | 77.766(2) |
| *γ* / ° | 90 | 76.153(3) |
| *V* / Å^3^ | 3038.16(16) | 3741.8(2) |
| *Z* | 4 | 2 |
| Independent reflections | 8864 | 15667 |
| *R* (*I* > 2*s*(*I*)) | R_1_ = 0.0306, wR_2_ = 0.0774 | R_1_ = 0.0472, wR_2_ = 0.1314 |
| *wR* (all data) | R_1_ = 0.0396, wR_2_ = 0.0799 | R_1_ = 0.0502, wR_2_ = 0.1341 |

**Table S1.** X-ray data for [Ir(ppy)_2_HYNIC] and [Ir(piq)_2_HYNIC]Cl•CH_3_CN•(CH_3_CH_2_)O

**4. Electronic Spectroscopy and Electrochemistry**

The quantum yields of all complexes were thus measured in both a deoxygenated acetonitrile^a^ and a deoxygenated solution of 1% DMSO in PBS (pH = 7.4)^b^ at room temperature. Dilute samples were deoxygenated by degassing by bubbling N_2_ and compared to known reference compound [Ru(bpy)_3_][Cl]_2_ ($\Phi=$ 0.094 in deaerated CH_3_CN and $\Phi=$0.062 in deoxygenated H_2_O), and the relative quantum yield calculated using the equation: $\Phi s=\Phi r(\frac{A_{r}}{A_{s}})(\frac{E_{s}}{E_{r}}){(\frac{n_{s}}{n_{r}})}^{2}$_._

|  | **Absorbance** | | **Emission** | | | | |
| --- | --- | --- | --- | --- | --- | --- | --- |
|  | λ_max_  (nm) | Ɛ  (M^-1^cm^-1^) | λ_ex/_λem  (nm) | Φem | τ  (µs) | K_r_  (x10^5^ s^-1^) | K_nr_  (x10^5^ s^-1^) |
|  |  |  |  |  |  |  |  |
| [Ir(ppy)­_2_HYNIC][PF_6_] | 255 | 42020 |  |  |  |  |  |
|  | 383 | 4060 | 380/485  (511 sh) | 0.26^a^  0.31^b^ | 2.58 | 1.01^a^ | 2.86^a^ |
|  |  |  |  |  |  |  |  |
| [Ir(piq)­_2_HYNIC][PF_6_] | 234 | 47840 |  |  |  |  |  |
|  | 289 | 33600 |  |  |  |  |  |
|  | 335(sh) | 15860 | 440/598 | 0.20^a^  0.009^b^ | 2.26 | 0.90^a^ | 3.52^a^ |
|  | 387(sh) | 7500 |  |  |  |  |  |
|  | 435 | 5620 |  |  |  |  |  |
|  |  |  |  |  |  |  |  |

Absorbance spectra were collected in acetonitrile (10 μM) and extinction coefficients for observed electronic transitions calculated using the Beer-Lambert law (Table S2).

**Table S2.** Absorption and emission maxima, quantum yields and fluorescence lifetimes.

**Fig. S13**. Fluorescence lifetime measurement for [Ir(ppy)_2_(HYNIC)]PF_6_.

**Fig. S14**. Fluorescence lifetime measurement for [Ir(piq)_2_(HYNIC)]PF_6_.

| **Compound** | **Oxidations (V) (E^1/2^) ^a,b^** | **Reductions (V) (E^1/2^)^a,b,c^** | | | |
| --- | --- | --- | --- | --- | --- |
| [Ir(ppy)_2_(HYNIC)][PF_6_] | 0.625^b^ |  | -2.50^b^ | -2.65^c^ | -2.94^c^ |
| [Ir(piq)_2_(HYNIC)][PF_6_] | 0.695^b^ |  | -2.09^b^ | -2.21^c^ | -2.44^c^ |
| [Ir(ppy)_2_(bpy)][PF_6_]**^d^** | 0.870 | -1.82 | -2.53^b^ | -2.64 | -2.95 |
| Assignment* | Ir^IV^/Ir^III^ |  | HYNIC | C^N | C^N |

**Table S3**. Summary of cyclic voltammetry data. Experiments conducted at 100 mV/s with 1 mM of analyte and 50 mM of NBu_4_PF_6_ as supporting electrolyte. b) Irreversible process c) Quasi reversible processes d) Values reported for [Ir(ppy)_2_(bpy)][PF_6_] under the same conditions and used as a reference. * C^N refers to the cyclometalating ligand (ppy or piq).

**Fig.** **S15.** Cyclic voltammograms for [Ir(ppy)_2_(HYNIC)]^+^ (a) and (b) and [Ir(piq)_2_(HYNIC)]^+^ (c) and (d).

**5. Investigation of reactive oxygen species generation**

**5.1. Determination of singlet oxygen generation quantum yields**

Air equilibrated solutions of [Ir(ppy)_2_(HYNIC)]PF_6_, [Ir(piq)_2_(HYNIC)]PF_6_ or [Ru(bpy)_3_]Cl_2_ (Φ_Δ_ = 0.57 in air equilibrated acetonitrile) in acetonitrile were adjusted in concentration such that their absorbance at λ = 365 nm was between 0.1 and 0.2. 1,3-diphenylisobenzofuran was added to each solution (10 mM final). Solutions were irradiated at λ = 365 nm and the absorbance at 411 nm monitored. The decrease in absorbance was plotted against irradiation time and the quantum yields of singlet oxygen generation determined using Equation 1. The photooxidation of DPBF was also measured and accounted for in the determination.

$$\Phi_{\Delta, sample}= \frac{M_{sample}}{M_{ref}}\times\frac{F_{ref}}{F_{sample}}\times\Phi_{\Delta,ref}$$

**Equation 1:** Φ_Δ_ is the quantum yield of singlet oxygen generation, M is the corrected slope of the linear fit of change in absorbance at 411 nm vs irradiation time, F = 1 – 10^AL^ (A = absorbance at λ = 365 nm and L = path length of cell in cm).

The corrected slopes are found by subtracting the slope of the linear fit of the change in absorbance at 411 nm vs irradiation time of the DPBF only from the slope of the linear fit of either the sample or the reference with 10 mM DPBF.

**Figure S16:** plot of A-A_0_ at 411 nm for DPBF (10 µM, blue) [Ir(ppy)_2_(pytr-NH_2_)] or [Ir(piq)_2_(pytr-NH_2_)] only (green), DPBF with [Ir(ppy)_2_(pytr-NH_2_)] or [Ir(piq)_2_(pytr-NH_2_)] (red), and DPBF with [Ir(ppy)_2_(pytr-NH_2_)] or [Ir(piq)_2_(pytr-NH_2_)] and 100 mM triethylamine (NEt_3_, quencher, black)

**5.2. Investigation of hydroxyl radical generation using hydroxyphenylfluorescein**

Solutions of either [Ir(ppy)_2_(HYNIC)]PF_6_ or [Ir(piq)_2_(HYNIC)]PF_6_ (10 μM) in phosphate buffered saline (PBS, pH 7.4) with 3’-(p-hydroxyphenyl) fluorescein (HPF) (5 μM) were irradiated (λ_exc_ = 420 nm, 26.84 mW/cm^2^) for the time intervals indicated and the fluorescence emission spectra measured (λ_exc_ = 490 nm) before and after irradiation. Increase in fluorescence intensity indicates the generation of hydroxyl radical. The same experiments were also carried out with terephthalic acid saturated PBS as a hydroxyl radical scavenger.

**Figure S17:** fluorescence emission spectra for hydroxyphenylfluorescein before and after irradiation with either [Ir(ppy)_2_(HYNIC)]PF_6_ (top left), [Ir(piq)_2_(HYNIC)]PF_6_ (top middle), hydroxyphenylfluorescein only (top right), [Ir(ppy)_2_(HYNIC)]PF_6_ and terephthalic acid (bottom left), [Ir(piq)_2_(HYNIC)]PF_6_ and terephthalic acid (bottom middle).

**5.3. Investigation of superoxide anion radical generation with dihydrorhodamine 123**

Solutions of either [Ir(ppy)_2_(HYNIC)]PF_6_ (10 μM) or [Ir(piq)_2_(HYNIC)]PF_6_ (10 μM) in phosphate buffered saline (pH 7.4) with dihydrorhodamine 123 (DHR) (5 μM) were irradiated (λ_exc_ = 420 nm, 26.84 mW/cm^2^) for the time intervals indicated and the fluorescence emission spectra measured (λ_exc_ = 500 nm) before and after irradiation. Increase in fluorescence intensity indicates the generation of superoxide anion radical. The same experiments were also carried out with tiron saturated PBS as a superoxide anion radical scavenger.

**Figure S18:** fluorescence emission spectra for dihydrorhodamine 123 before and after irradiation with either [Ir(ppy)_2_(HYNIC)]PF_6_ (top left), [Ir(piq)_2_(HYNIC)]PF_6_ (top middle), dihydrorhodamine 123 only (top right), [Ir(ppy)_2_(HYNIC)]PF_6_ and tiron (bottom left), [Ir(piq)_2_(HYNIC)]PF_6_ and tiron (bottom middle).

**6. Log *D*_7.4_ determination for HYNIC-PSMA complexes**

1 μL of a 0.015 M stock solution of either [Ir(ppy)_2_(HYNIC-PSMA)] or [Ir(piq)_2_(HYNIC-PSMA)] in dimethyl sulfoxide, 299 μL octanol saturated PBS and 300 μL PBS saturated octanol were vortexed for 10 mins followed by 2 h centrifugation at 14 000 rcf. The layers were separated and absorbance at 280 nm ([Ir(ppy)_2_(HYNIC-PSMA)]) or 460 nm ([Ir(piq)_2_(HYNIC-PSMA)]) was used to calculate log D_7.4_ according to Equation 2. Log *D*_7.4_ = -0.22 for [Ir(ppy)_2_(HYNIC-PSMA)], and -0.32 for [Ir(piq)_2_(HYNIC-PSMA)].

$$\log D7.4=\log_{10} \frac{A_{octanol}}{A_{PBS}}$$

**Equation 2:** equation used for determining log *D*_7.4_. A_octanol_ is the absorbance at the selected wavelength of the PBS saturated octanol layer, and A_PBS_ is the absorbance at the selected wavelength of the octanol saturated PBS layer.

**7. Cell culture**

LNCaP cells were cultured in RPMI 1640 media supplemented with 10% fetal bovine serum, at 37 ºC in a 5% CO_2_ atmosphere. Cells were passaged and all cellular experiments carried out according to standard aseptic procedures.

**8. Live cell confocal microscopy**

Cells in growth medium were seeded (60 000 cells/well) into poly-L-lysine treated wells of 8-well chamber slides (ibidi) and incubated at 37 ºC in a 5% CO_2_ atmosphere for 18 – 48 h to adhere. Cells were then incubated with [Ir(piq)_2_(HYNIC-PSMA)]^+^ (25 μM))]^+^ in complete growth medium. Cells for the autofluorescence control did not receive this treatment step. Cells were washed twice with DPBS then media changed into phenol red free RPMI 1640 media supplemented with 10% fetal bovine serum and Hoescht 33342 (20 µg/mL) before imaging with a Zeiss Elyra LSM880 with a 40x air objective lens. Excitation wavelengths λ = 488 nm as well as λ = 405 nm. During imaging cells were incubated at 37 ºC in a 5% CO_2_ atmosphere. Images were processed in ImageJ and brightness and contrast adjusted uniformly across all images.


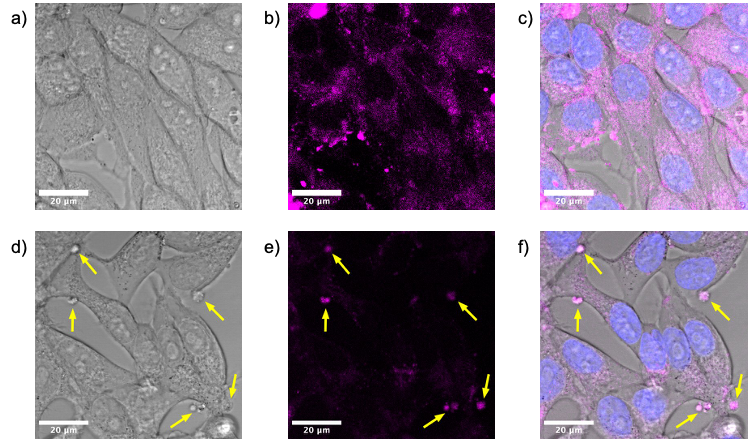


**Figure S19:** Confocal microscope images of LNCaP cells treated with Hoescht 33342 (nuclear stain, blue, λ_ex_ = 405 nm) with (a-c) or without [Ir(piq)_2_(HYNIC-PSMA)]^+^ (d-f) for an ‘autofluorescence control’. Left: brightfield image. Middle: λ_ex_ = 488 nm channel only. Right: λ_ex_ = 405 nm, λ_ex_ = 488 nm, and brightfield images overlaid. Arrows indicate cell debris visible on brightfield image that auto fluoresces (middle and right; λ_ex_ = 488 nm channel).

**9. Cytotoxicity and phototoxicity assays**

Cell cytotoxicity and photocytotoxicity assays were carried out in black-walled, UV-clear flat bottomed 96-well plates that had been polylysine treated. LNCaP cells were seeded into the inner wells (20 000 cells/well) in complete growth medium, leaving one column without cells. Peripheral wells had 200 μL DPBS/well throughout the entire assay to minimise evaporation from inner wells. Cells were allowed to adhere overnight at 37 ºC in a 5% CO_2_ atmosphere. Media was removed and cells were incubated in RPMI 1640 medium supplemented with 1% fetal bovine serum for 1 h at 37 ºC in a 5% CO_2_ atmosphere. [Ir(ppy)_2_(HYNIC-PSMA)], [Ir(piq)_2_(HYNIC-PSMA)], [Ir(ppy)_2_(HYNIC)]Cl or [Ir(piq)_2_(HYNIC)]Cl were serially diluted in complete growth and added to the appropriate wells, with n = 3 for each concentration. Controls were cells treated with complete growth medium only (100% viability) and wells with no cells treated with complete growth medium only (0% viability). Cells were incubated for 1 h at 37 ºC in a 5% CO_2_ atmosphere before each well was washed with 3 x 100 μL DPBS. Cells were either kept in the dark at 37 ºC in a 5% CO_2_ atmosphere or irradiated (λ_exc_ = 420 nm, 30 minutes, 26.84 mW/cm^2^). If cells were to be irradiated, they were media changed into phenol red free RPMI 1640 medium supplemented with 10% fetal bovine serum after washing and before irradiation. Cells were then media changed into fresh RPMI 1640 medium supplemented with 10% fetal bovine serum and incubated overnight at 37 ºC in a 5% CO_2_ atmosphere. 10 μL of MTT solution (5 mg/mL in DPBS) was added to each well and the plate incubated for 2 h at 37 ºC in a 5% CO_2_ atmosphere. 100 μL solubilising solution (0.01% HCl, 10% Triton X-100, 10 % DMSO in isopropanol) was added to each well and the plate incubated overnight at 37 ºC. Wells were thoroughly mixed and 90 μL from each well transferred into a new UV-clear 96-well plate. The absorbance of each well at λ = 570 nm and λ = 690 nm was measured.

**Figure S20:** concentration vs. cell viability with (red) and without (blue) irradiation for [Ir(ppy)_2_(HYNIC-PSMA)] (left), [Ir(ppy)_2_(HYNIC)]Cl (middle), and [Ir(piq)_2_(HYNIC)]Cl (right).

**10. Competitive binding assay**

Binding affinity of the HYNIC-PSMA conjugates to the PSMA enzyme was determined with a competitive binding assay in PSMA-expressing LNCaP cells.

24-well cell culture plates were coated with poly-L-lysine. 150,000 LNCaP cells were seeded the day before the experiment. Experiments were carried out in triplicate for each concentration. The RPMI-1640 culture medium was removed, and the cells were washed once with assay buffer (500 μL, Hank's balanced salt solution, with 1% BSA) before being left to equilibrate on ice for 15 min (200 μL, assay buffer). 25 μL/well of either assay buffer (control) or of solutions containing either [Ir(ppy)_2_(HYNIC-PSMA)] or [Ir(piq)_2_(HYNIC-PSMA)] in increasing concentrations (10^-10^-10^-4^ M in assay buffer) were added, followed by the addition of [^177^Lu][LuPSMA-617]^[8]^ (of 25 μL, SA = 15 MBq/nmol in HBSS (1% BSA). The final ([^177^Lu]LuPSMA-617 concentration was 0.35 nM in all binding assays. Cells were incubated on ice for 60 min. Incubation was terminated by the removal of the incubation medium. Cells were thoroughly rinsed with 250 μL of HBSS. The wash medium was combined with the supernatant of the previous step. This fraction represents the amount of free ([^177^Lu]LuPSMA-617. Cells were then lysed using 250 μL of 1 N NaOH, incubated for 15 minutes, and the lysate was transferred to vials and combined with 250 μL of HBSS used for rinsing the wells. Quantification of the amount of free and bound activity was performed in a γ-counter (Revvity, wizard). IC_50_ values were 6.2 nm for [Ir(ppy)_2_(HYNIC-PSMA)] and 8.5 nm for [Ir(piq)_2_(HYNIC-PSMA)].

**Figure S21:** Percentage [^177^Lu]LuPSMA-617 bound vs. concentration of either [Ir(ppy)_2_(HYNIC-PSMA)] (blue) or [Ir(piq)_2_(HYNIC-PSMA)] (red).

**5. References**

[1] A. M. Brouwer, *Pure Appl. Chem.* **2011**, *83*, 2213-2228.

[2] S. Sprouse, K. A. King, P. J. Spellane, R. J. Watts, *Journal of the American Chemical Society* **1984**, *106*, 6647-6653.

[3] M. J. Abrams, M. Juweid, C. I. tenKate, D. A. Schwartz, M. M. Hauser, F. E. Gaul, A. J. Fuccello, R. H. Rubin, H. W. Strauss, A. J. Fischman, *Journal of Nuclear Medicine* **1990**, *31*, 2022.

[4] N. A. Zia, C. Cullinane, J. K. Van Zuylekom, K. Waldeck, L. E. McInnes, G. Buncic, M. B. Haskali, P. D. Roselt, R. J. Hicks, P. S. Donnelly, *Angew. Chem., Int. Ed.* **2019**, *58*, 14991-14994.

[5] G. M. Sheldrick, *Acta Crystallogr., Sect. C Struct. Chem.* **2015**, *71*, 3-8.

[6] O. V. Dolomanov, L. J. Bourhis, R. J. Gildea, J. A. K. Howard, H. Puschmann, *J. Appl. Crystallogr.* **2009**, *42*, 339-341.

[7] C. F. Macrae, I. Sovago, S. J. Cottrell, P. T. A. Galek, P. McCabe, E. Pidcock, M. Platings, G. P. Shields, J. S. Stevens, M. Towler, P. A. Wood, *J. Appl. Crystallogr.* **2020**, *53*, 226-235.

[8] M. Benešová, M. Schäfer, U. Bauder-Wüst, A. Afshar-Oromieh, C. Kratochwil, W. Mier, U. Haberkorn, K. Kopka, M. Eder, *J. Nucl. Med.* **2015**, *56*, 914.
